# An Energetic-Control Framework for Electrospray Ionization Using Thermometer Ions

**DOI:** 10.1021/jasms.6c00109

**Published:** 2026-10-01

**Authors:** Max T. G. M. Derks, Jana Roithová

**Affiliations:** Institute for Molecules and Materials, Radboud University, Heyendaalseweg 135, Nijmegen 6525 AJ, The Netherlands

**Keywords:** Electrospray ionization, Thermometer ions, Internal energy calibration, Design of experiments, Ion activation, Survival yield, Flow rate effects

## Abstract

Electrospray ionization mass spectrometry (ESI-MS) is increasingly used to investigate chemical reactivity. During these experiments, source conditions are frequently adjusted, either deliberately or as part of routine method optimization. However, the energetic consequences of these changes are rarely known, meaning that experiments may unknowingly be performed under different energetic conditions. Consequently, differences observed between experiments may reflect changes in ion activation rather than changes in solution or droplet chemistry, particularly when reactive intermediates or labile species are investigated. Here, we establish a practical, source-specific energetic-control framework using thermometer ions to provide an independent relative energetic reference for a defined electrospray source. A multivariate Design of Experiments revealed that the energetic landscape is governed by strong interactions between source parameters, making the energetic consequences of changing source settings difficult to anticipate and potentially variable between nominally similar experiments. The framework also identified operating regimes in which differential ion transmission distorted the apparent energetic response. After identifying and excluding these transmission-distorted regions, the remaining energetic landscape was accurately described by a predictive multivariate model. The resulting framework provides an independent energetic reference that can be established alongside chemical measurements to distinguish source-induced energetic effects from genuine chemical transformations. We therefore propose that, analogous to routine mass calibration, the energetic response of an electrospray source should be experimentally characterized whenever ion internal energy may influence the observed chemistry.

## Graphical abstract

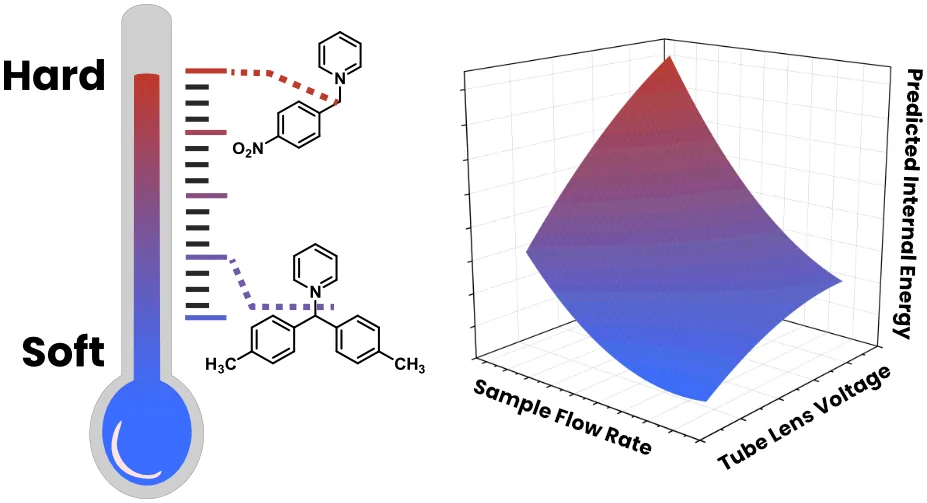

## Introduction

The terms “soft” and “hard” ionization are widely used in mass spectrometry (MS) to describe the extent of fragmentation imparted during ion formation. In electrospray ionization (ESI), where intact molecular ions are typically desired, the “softness” of the ionization process is often assumed rather than quantitatively assessed.^1,2^ Nevertheless, source parameters such as capillary temperature, tube-lens voltage, capillary voltage, gas flows, and source geometry are routinely adjusted to optimize analytical performance.^3–7^ (Note that source geometry depends on the mass spectrometer manufacturer and, accordingly, equivalent source potentials may have different names; e.g., cone voltage.) Source parameters are also deliberately varied outside conventional MS optimization.

In flow-chemistry experiments, the sample flow rate is commonly adjusted to control solution-phase reaction time.^8,9^ In studies of in-droplet reactivity and interface reaction acceleration, parameters including sample flow rate, source distance, and capillary temperature are frequently varied because they influence droplet size, droplet lifetime, evaporation rate, and consequently the extent of reaction occurring within electrosprayed droplets.^10–12^ As ESI-MS increasingly evolves from a purely analytical technique into a platform for investigating reactive systems, the energetic consequences of changing source conditions become increasingly important to quantify. Without an independent energetic reference, source-induced ion activation may become an unrecognized confounding factor in the interpretation of measured reaction outcomes. Among these parameters, sample flow rate exemplifies this challenge. While it is commonly adjusted to control reaction time in flow chemistry, it also alters droplet formation, evaporation, and desolvation.^13,14^ Consequently, changing the flow rate has the potential to influence not only the intended experimental conditions but also the ionization process through which the experiment is ultimately observed.

Several studies have reported that varying the sample flow rate substantially changes the observed mass spectrometric response. Bristow et al. showed that increasing the sample flow rate reduced in-source fragmentation across several ESI platforms, which they attributed to higher partial pressures and greater collisional cooling in the source region.^15^ While this work clearly demonstrated that source conditions can substantially alter ion activation, the analysis relied on fragment intensity ratios and spectral matching metrics rather than explicitly quantifying the underlying changes in gas-phase internal energy. Similarly, Pedro et al. observed changes in measured protein–ligand equilibrium dissociation constants as a function of solution flow rate.^16^ Likewise, Di Marco et al. reported enhanced detection of AlL_3_ dimeric species under conditions promoting larger droplets.^17^ Collectively, these studies demonstrate that changes in sample flow rate can substantially alter the observed analytical outcome. However, because these same conditions also modify the energetic state of the detected ions, it often remains unclear to what extent the observed changes originate from genuine chemistry, source-induced ion activation, or a combination of both. An independently measured energetic reference is therefore required to separate these contributions.

Thermometer ions provide a direct route to this missing energetic information. These ions undergo predictable fragmentations governed by well-defined bond dissociation energies (BDEs)^18–20^ and can be used to infer internal-energy distributions from their survival yields.^21,22^ Benzylpyridinium and benzhydrylpyridinium ions are among the most widely used thermometer ions for characterizing ion activation and comparing ionization environments.^1,3,19–21,23,24^ For example, Kaeslin et al. used benzylammonium thermometer ions to benchmark SESI-MS against ESI-MS,^1^ while Lecchi et al. proposed thermometer-ion survival-yield vectors and Euclidean distance metrics as a practical tool for monitoring ESI reproducibility.^21^ Although these studies demonstrate the utility of thermometer ions for characterizing ionization conditions, they did not combine them with multivariate experimental designs to establish predictive energetic models or investigate how multiple source parameters collectively determine ion activation.

The objective of this work is to establish a practical, source-specific energetic control by constructing a relative predicted mean internal-energy axis. This energetic axis provides an independent reference against which reaction transformations measured under matching source conditions, including studies of reaction acceleration in electrosprayed droplets, can subsequently be interpreted, allowing the energetic contribution of source-induced ion activation to be evaluated alongside the observed chemical response.

The resulting energetic axis is not intended as a universally transferable or absolute thermodynamic calibration, but as a consistent relative coordinate for a defined source and solution system. For this application, we map the survival yields of the thermometer ions on their theoretical bond dissociation energies (*E*_0_ values). More accurately, the theoretical *E*_0_ values should be converted to the expected appearance energies (*E*_app_) by RRKM modeling to account for the possible kinetic shift.^25^ This conversion would, however, require a defined activation time scale,^25^ which is unknown and likely to vary across the investigated in-source conditions. Hence, the *E*_0_-based energy scale can underestimate absolute internal energies. Nevertheless, it provides a suitable operational reference scale for mapping and correlating relative energetic changes.

To construct this energetic-control framework, we combined a mixture of benzylpyridinium and benzhydrylpyridinium thermometer ions with a multivariate Design of Experiments (DoE). Because the investigated source parameters were expected to interact strongly, we used a DoE to determine which experimental parameters move the system along the energetic axis. Although DoE has previously been applied to optimize ESI operating conditions,^16,22,26^ it is used here for a different purpose. Rather than treating the source as a black-box optimization problem, the DoE is used to quantify the direction, magnitude, interactions, and nonlinearities of the source-parameter effects on ion activation. In doing so, the DoE not only captures the complexity of the electrospray source but also demonstrates that its energetic response is sufficiently systematic to be described by a predictive multivariate model. Sample flow rate, capillary temperature, sheath-gas flow rate, spray voltage, and capillary voltage were selected as representative source parameters for constructing the framework.

Finally, each experimental condition was acquired across a tube-lens voltage gradient rather than at a single fixed setting. This proved essential for identifying regions in which differential ion transmission distorts the thermometer-ion response and could otherwise be mistaken for changes in internal energy. The Results and Discussion therefore first establish the need for identifying and excluding these transmission-distorted regimes before presenting the resulting source-specific multivariate model.

## Methodology

The thermometer ions used in the analysis include *p*-H,H, *p*-OMe,OMe, *p*-Me,Me, *p*-NO_2_, *p*-CN, *p*-CH_3_, *p*-OCH_3_, and *p*-F derivatives of benzylpyridinium ions. The *E*_0_ values were obtained from a recently reported review.^23^ Note that for *p-*NO_2_, the extracted ion traces were corrected for a secondary fragmentation pathway. These compounds were either available from previous work in our laboratory or synthesized according to the previously reported literature procedures.^27,28^

### Sample Preparation

Stock solutions (1 mg mL^–1^) of each thermometer ion (benzylpyridinium and benzhydrylpyridinium derivatives) were prepared in 1:1 (v/v) methanol–water. For each experiment, 100 μL of stock solution was transferred to a vial and diluted to a final volume of 10 mL with 1:1 methanol–water to yield the working solution. The working solution (thermometer ion mix) was loaded into a 1002 TLL syringe (Hamilton, Reno, NV) and was infused using a syringe pump (Labm8 M8003, Labm8, Netherlands). The stepper motor was operated at 256 microsteps unless otherwise stated Table 1.

**Table 1:** Thermometer Ion Used in the Analysis, with the Used Masses and Energy Levels as Recently reported^23^

Thermometer ion	Precursor mass	Fragment mass	*E*_0_ (eV), CCSD(T)
Benzylpyridinium			
*p-CH*_3_	184.1	105	2.267
*p-OCH*_3_	200.1	121	1.86
*p-F*	188.1	109	2.392
*p-NO*_2_	215, 169	136	2.843
Benzhydrylpyridinium			
*p-H,H*	246.1	167	1.74
*p-OCH*_3_,*OCH*_3_	305.5	227	1.18

### Experimental Design

Source parameters were investigated using a full-factorial design comprising 72 nominal experimental conditions (2 × 3 × 2 × 2 × 3 levels). The tube-lens voltage was not included as a factorial parameter but was instead scanned as a stepwise gradient at each factorial design point, resulting in 792 unique measurement conditions. To mitigate systematic drift and thermal equilibration effects, experiments were blocked by capillary temperature and, within each temperature block, by sample flow rate. Runs were randomized within these blocks.

All measurements for the DoE were performed on a Thermo Scientific LTQ XL linear ion trap. Measurements shown in Figure 10 were acquired on a Thermo TSQ Classic instrument. Offline sample flow rate measurements were made using a Sensirion SLF3S-0600F liquid flow sensor. The Design of Experiments comprised six representative source parameters expected to define the energetic landscape of the Thermo LTQ source. Their investigated levels are listed in Table 2, while their approximate location within the source is illustrated schematically in Figure 1.

**Table 2:** Parameters and Levels Tested in the DoE

Parameter	Levels
Spray Voltage (kV)	3.5, 5
Capillary Temperature (°C)	150, 220, 300
Sheath Gas (arb. units)	5, 20
Capillary Voltage (V)	40, 100
Sample Flow rate (μL min^–1^)	5, 25, 80
Tube Lens Voltage (V)	40–240 (20 V increments)

**Figure 1:**
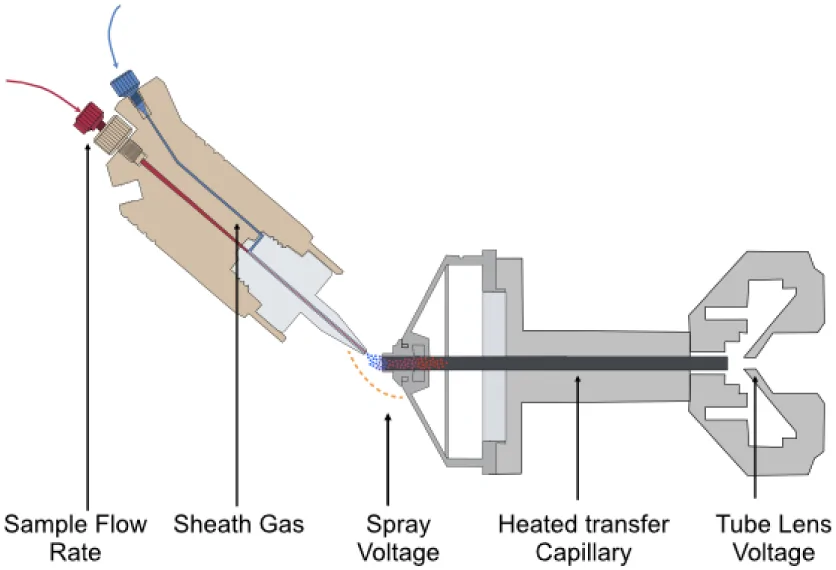
Schematic representation of the Thermo LTQ electrospray ionization source showing the location of the source parameters investigated in the Design of Experiments. The drawing is not to scale.

All source parameters were manually configured on the mass spectrometer. The tube lens voltage gradient was controlled via an AutoIt script that incremented the voltage in 20 V steps at 20 s intervals.

To ensure consistency between the designed DoE matrix and the executed experiments, a dedicated data-processing script was developed. This script parsed the RAW files and extracted instrument metadata for each acquisition, allowing automated cross-verification against the predefined DoE table.

### Data Processing

At each scan time point, precursor and fragment ion intensities were extracted for each thermometer by integrating the signal within ±0.5 *m*/*z* of the target ion. Survival yields (SY) were calculated as

$$SY=\frac{I_{\mathrm{surv}}}{I_{\mathrm{surv}}+\sum I_{\mathrm{frag}}}$$

where *I*_surv_ is the precursor intensity (and, where applicable, additional “pseudo-precursor” channels counted as surviving signal) and $\sum I_{\mathrm{frag}}$ is the summed intensity of the corresponding fragment channel(s). For each condition, mean SY values were plotted as a function of the thermometer-ion critical energies (*E*_0_, Table 1), obtained from literature CCSD­(T) bond dissociation energies, and fitted with a logistic function of the form:

$$SY\left(E\right)=\frac{1}{1+\exp\left(-\frac{E-x_{0}}{B}\right)}$$

Fits were obtained by minimizing the squared error using a deterministic grid search over *B* and *x*_0_. To stabilize fits in regimes with partially saturated survival yields and to constrain extrapolation outside the available *E*_0_ window, two anchor points at (0, 0) and (5, 1) were included with fixed weights. The parameter *x*_0_ defines the inflection point of the survival-yield curve, corresponding to both the midpoint of the transition (SY = 0.5) and the maximum of the first derivative (*d*SY/*dE*). It was used to represent the relative mean internal energy of each experimental condition, while *B* describes the width of the transition. These relative mean internal-energy values were subsequently grouped by tube-lens voltage and averaged in 20 V increments (tube-lens bins obtained by rounding to the nearest 20 V).

The use of thermometer-ion critical energies (*E*_0_) as the reference energy axis and the interpretation of the SY–*E*_0_ inflection point as an operational energetic reference follow established survival-yield methodologies.^1,2,18,23,29^ The resulting energetic axis should be interpreted as a source-specific relative coordinate for comparing energetic changes under matched experimental conditions, rather than as an absolute thermodynamic measure of ion internal energy.^18,23^

Multivariate modeling of internal energy was performed in JMP Student Edition 19.0.1 (JMP Statistical Discovery LLC, Cary, NC, USA) using the Fit Model platform (Standard Least Squares). The inferred internal energy values (x_0_) were used as the response variable. Model terms were generated using the built-in response surface macro, yielding a second-order polynomial model including main effects, two-factor interactions, and quadratic terms. Terms were evaluated based on statistical significance (α = 0.05) and overall model performance (R^2^ and adjusted R^2^). Model reduction followed hierarchical principles, such that lower-order terms were retained when associated higher-order interactions or quadratic terms were statistically significant. Although weighting strategies based on fit uncertainty were explored during model development, the final models were constructed without weighting, as this provided more stable and interpretable parameter estimates across the full experimental range.

## Results and Discussion

Figure 2 shows the inferred internal energy as a function of tube-lens voltage for a representative DoE condition (run 3), in which all source parameters except the tube-lens voltage were held constant. While increasing the tube-lens voltage is commonly assumed to monotonically increase ion activation, the data instead reveal a pronounced nonlinear response characterized by a distinct maximum in inferred internal energy.

**Figure 2:**
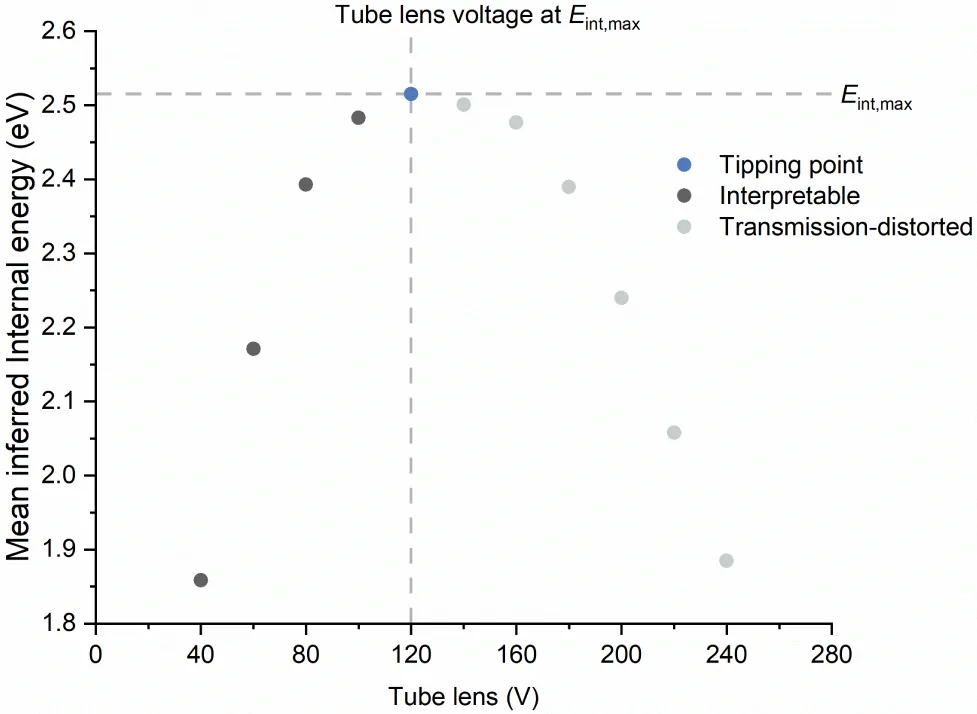
Mean internal energy inferred from thermometer-ion calibration as a function of tube-lens voltage for a representative DoE run. The maximum observed internal energy (*E*_int,max_) was used to identify the onset of transmission-limited behavior. Data points preceding *E*_int,max_ were considered energetically interpretable, whereas subsequent points were considered transmission-distorted and excluded from further analysis.

Beyond this maximum, further increases in tube-lens voltage led to an apparent decrease in inferred internal energy, despite continued increases in voltage that accelerate the ions. This behavior defines a tipping point in the tube-lens response, separating an energy-sensitive regime from a transmission-limited regime.

Figure 3 shows the mean fragment intensities, precursor intensities, and resulting survival yields as a function of tube-lens voltage for the same DoE condition. Fragment ion intensities initially increase with tube-lens voltage, reach a maximum, and subsequently decrease at higher voltages. Importantly, this decrease occurs in a regime where precursor ion intensities are already substantially depleted. As a consequence, fragment ions are lost from an already reduced precursor pool, leading to an apparent inversion of the survival yield. Under these conditions, fitting a logistic model to the survival yield no longer provides a reliable measure of internal energy.

**Figure 3:**
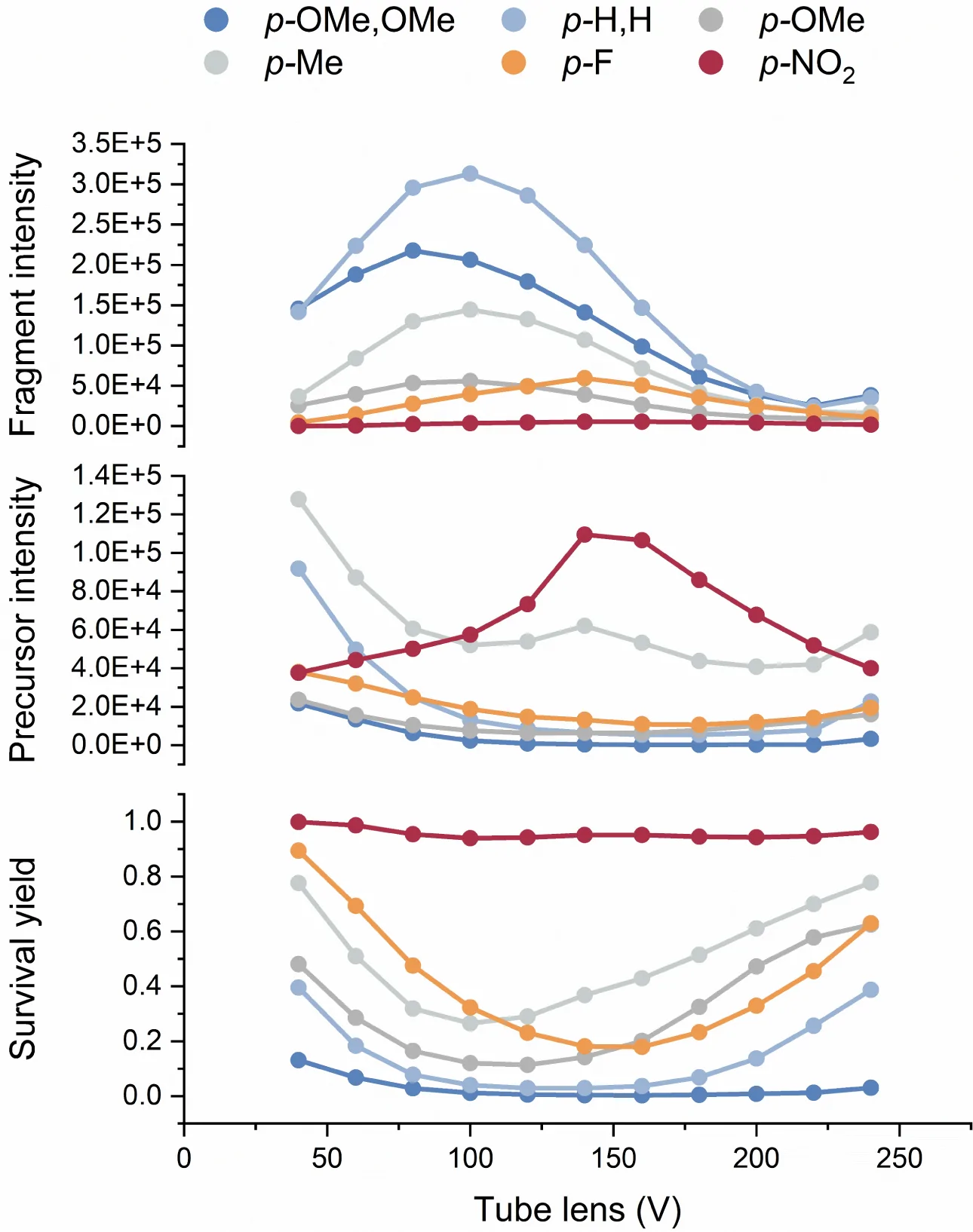
Mean fragment intensities (top), precursor intensities (middle), and survival yields (bottom) as a function of tube-lens voltage, averaged over binned tube-lens settings for the thermometer-ion series. Differential transmission losses at elevated tube-lens voltages disproportionately reduce fragment intensities relative to precursor ions, leading to an inversion in survival yield behavior.

This behavior is inconsistent with reduced ion activation and instead indicates transmission-limited conditions. Control experiments using 2,6-lutidine, a structurally robust probe molecule, show a comparable decrease in signal intensity at elevated tube-lens voltages, with no detectable fragment ions. This observation supports an ion-optical transmission origin for the observed signal loss (Figure S1).

Having established the origin of the tube-lens tipping point for a single DoE condition, we next examined how its occurrence varies across the full design-of-experiments space (Figure 4).

**Figure 4:**
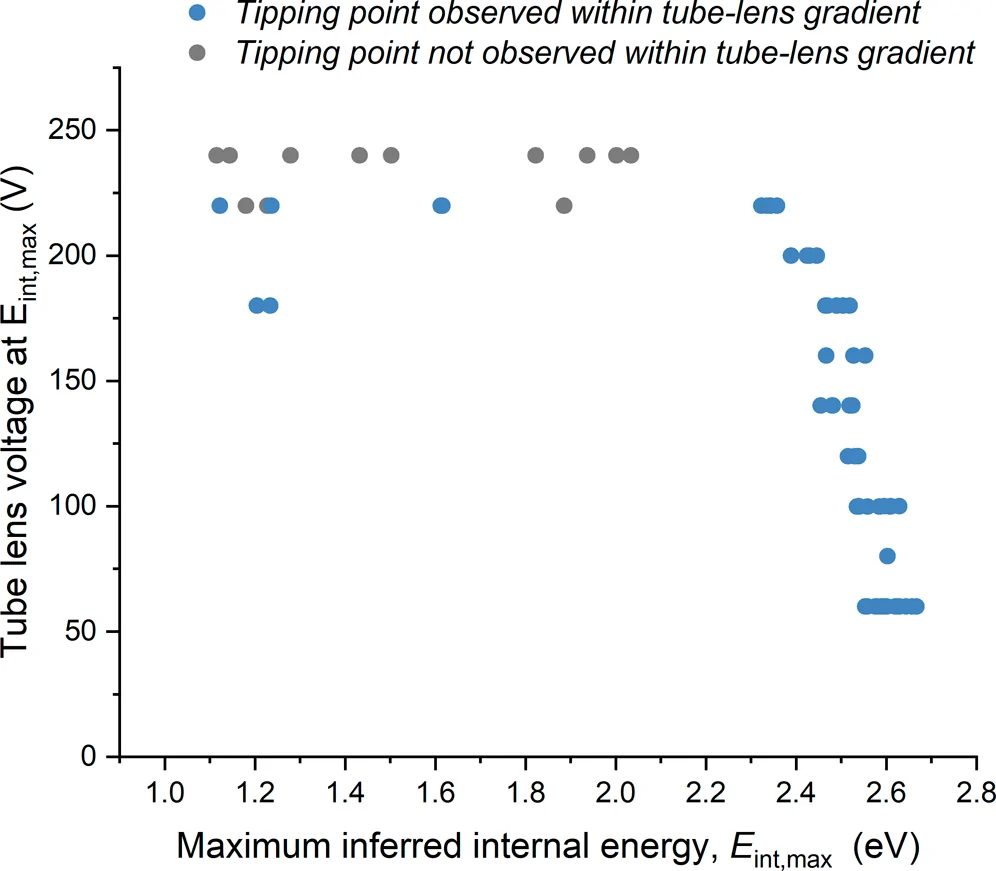
Distribution of tube-lens voltages corresponding to the maximum inferred internal energy (*E*_int,max_) across the experimental design space. Blue symbols indicate experiments in which a maximum inferred internal energy *E*_int,max_ was reached within the investigated tube-lens gradient. Gray symbols indicate experiments in which no maximum was observed before the upper tube-lens voltage limit was reached. Tube-lens gradient runs 20 and 42 were excluded from the analysis because they produced poor-quality data.

The tube-lens voltage at which a tipping point occurs varies strongly across the DoE space. In some runs, a tipping point occurs well within the scanned tube-lens range, whereas in others, no tipping point is reached, indicating that all measurements remain within the energy-sensitive regime across the investigated voltages. As a result, identical tube-lens settings can correspond to the rising, optimal, or declining region of the “energy–response curve” depending on the other source conditions.

Importantly, conventional data-quality control metrics—such as the goodness-of-fit of the logistic survival-yield model (Figure S2)—do not capture this regime ambiguity. This behavior complicates the construction of a design of experiments that probes the full instrumental parameter space. Measurements with unrecognized ion-transfer suppression, sampling the skewed “energy–response curve”, would provide *skewed configurations*. Therefore, we retained only measurements acquired within the energy-sensitive regime, before the tube-lens tipping point (dark gray points, Figure 2).

Having established a physically well-defined regime in which thermometer ions reliably report internal energy, we next quantitatively modeled the remaining data. Using the filtered data set, a response-surface model was constructed to describe the dependence of mean internal energy on ESI source parameters and fitted using standard least-squares regression. Tube-lens voltage was sampled at 11 levels, sample flow rate and temperature at 3 levels each, and spray voltage and capillary voltage at 2 levels. Consequently, quadratic terms were estimable only for tube lens, flow rate, and temperature, while spray voltage and capillary voltage were modeled as linear factors. The fitted model was subsequently reduced by removing insignificant higher-order terms while retaining lower-order main effects involved in significant interactions. The resulting model explains a substantial fraction of the observed variance (R^2^ ≈ 0.87) with an RMSE of 0.143 eV (Figure 5). However, a clear S-shaped deviation is observed in the measured-versus-predicted plot. The deviations occurring above 2.4 eV and below 1.5 eV arise from the limited ability of the thermometer-ion framework to reliably report internal energies outside this range. In these regions, the internal energies lie outside the effective and experimentally accessible bond-dissociation energy window of the available thermometer ions. Consequently, only a small number of ions contribute survival yields within the informative (nonsaturated) region of the survival curve, and the inferred internal energy becomes predominantly constrained by the anchor points rather than by redundant thermometer-ion information.

**Figure 5:**
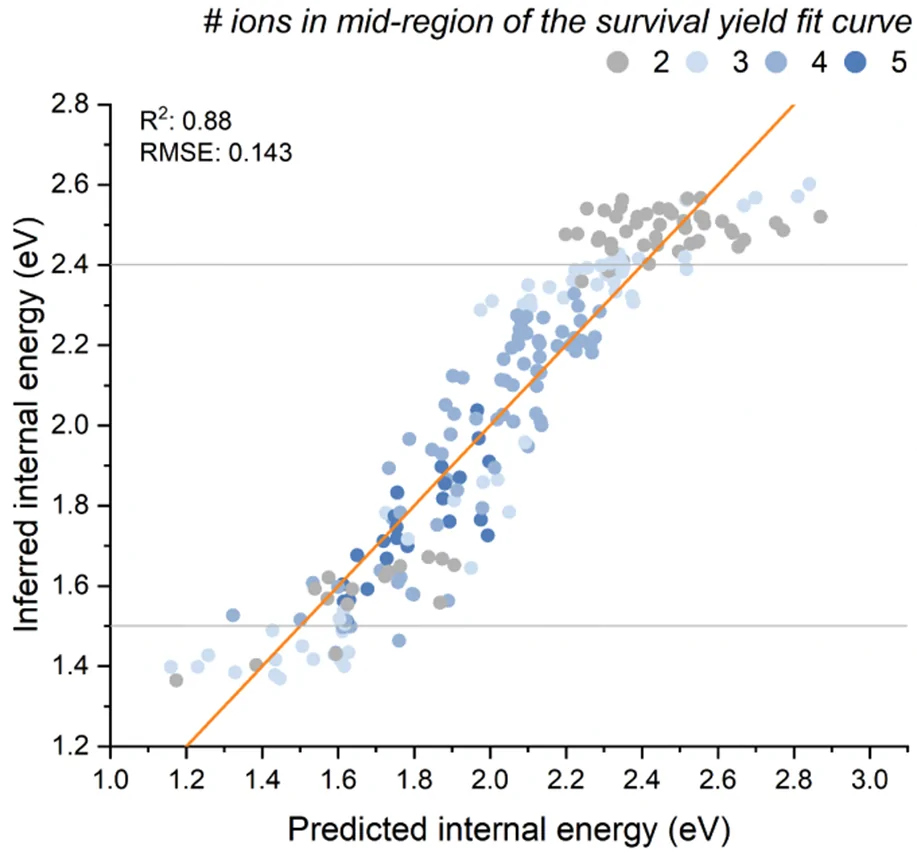
Measured versus predicted mean internal energy. Points are colored by the number of thermometer ions with nonsaturated mean survival yields $\left(0.15<\overset{↼}{SY}<0.85\right)$ in the corresponding Tube Lens bin. Deviations from the 1:1 line at low and high energies coincide with reduced thermometer-ion redundancy, while near-linear agreement is observed in the central energy range (1.5–2.4 eV).

The analysis in Figure 5 shows that systematic deviations in the global model occur when thermometer-ion survival yields saturate, and the number of informative ions decreases. Accordingly, the analysis was restricted to the linear response regime (1.5–2.4 eV, gray reference bands in Figure 5), which removes the observed curvature and improves the prediction error (RMSE reduced from 0.143 to 0.110 eV) while maintaining comparable explained variance (R^2^ ≈ 0.87). Furthermore, no major changes in the effect structure were observed upon restricting the analysis to the linear regime (see SI), as the relative ordering and statistical significance of the dominant main effects and interactions remained largely unchanged. The complete mathematical expressions of both reduced multivariate models are provided in the Supporting Information (Equations S1 and S2).

The combination of the tipping-point analysis and the restriction to the linear thermometer-ion regime yields a response-surface model that more closely reflects underlying changes in ion activation. The surface is described by an equation relating the investigated source parameters to the predicted internal energy. This derived equation can be used to perform virtual experiments. Accordingly, individual parameters were varied while all remaining parameters were held at the midpoint of their investigated experimental ranges. The resulting main-effect trends, shown in Figure 6, therefore reflect the isolated influence of each variable on the model-predicted internal energy.

**Figure 6:**
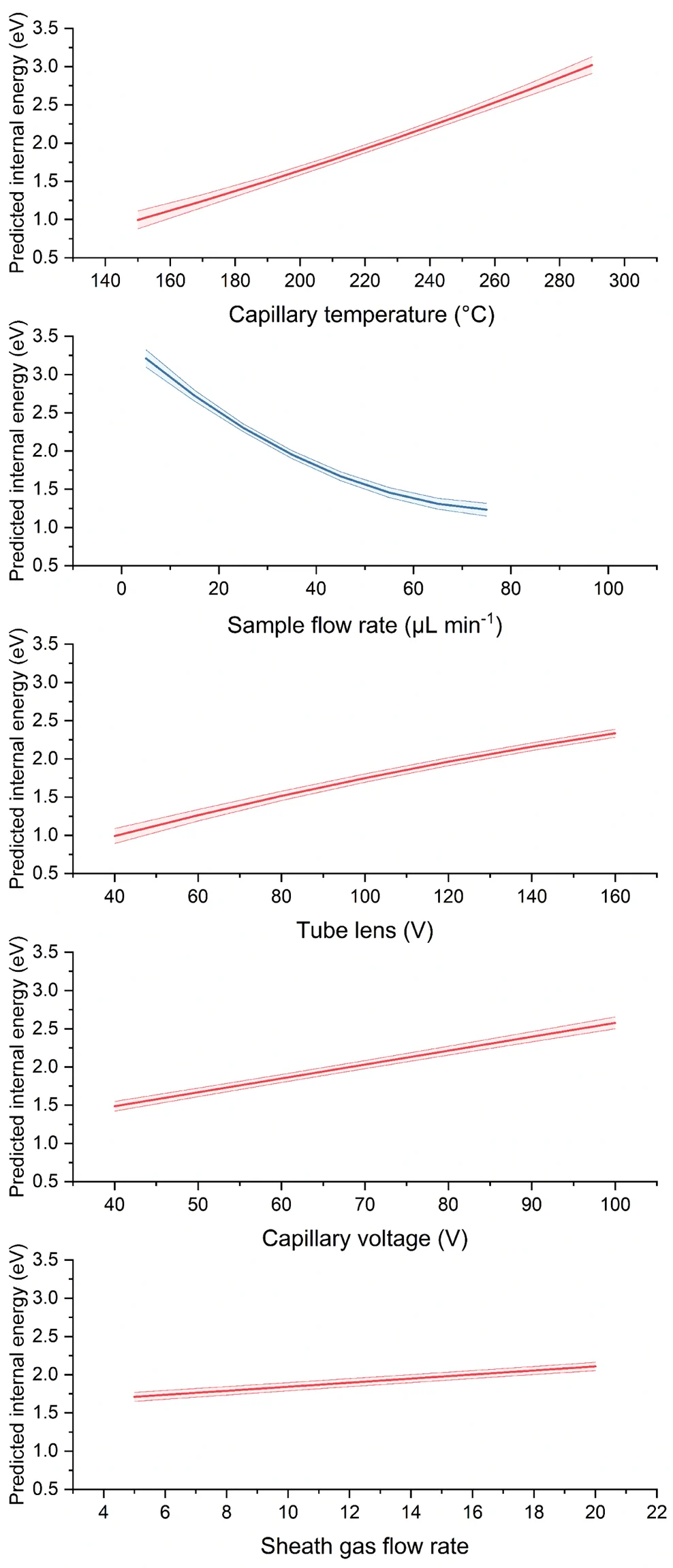
Main effects of capillary temperature, sample flow rate, tube-lens voltage, capillary voltage, and sheath-gas flow on the predicted mean internal energy in the linear response regime (1.5–2.4 eV). Lines show model predictions with all other factors held at their mean values; shaded regions indicate the 95% confidence interval.

Capillary temperature, tube-lens voltage, and capillary voltage each show a positive monotonic relationship with internal energy, whereas increasing sample flow rate produces a pronounced opposing trend. Over the explored parameter space, the magnitude of the flow rate effect is comparable to that of the thermal and electrical parameters.

While these main effects capture the dominant trends described by the model, several interaction and quadratic terms were also statistically significant. Figure 7 provides a schematic overview of the significant interactions and quadratic effects identified in the fitted model. Interactions involving thermal parameters and interface voltages (e.g., capillary temperature × capillary voltage and capillary temperature × tube-lens voltage), as well as interactions between sample flow rate and interface voltages, are among the most statistically significant. Line thickness reflects the relative statistical significance (logworth) of each term. A complete listing of all effects and model coefficients is provided in the Supporting Information.

**Figure 7:**
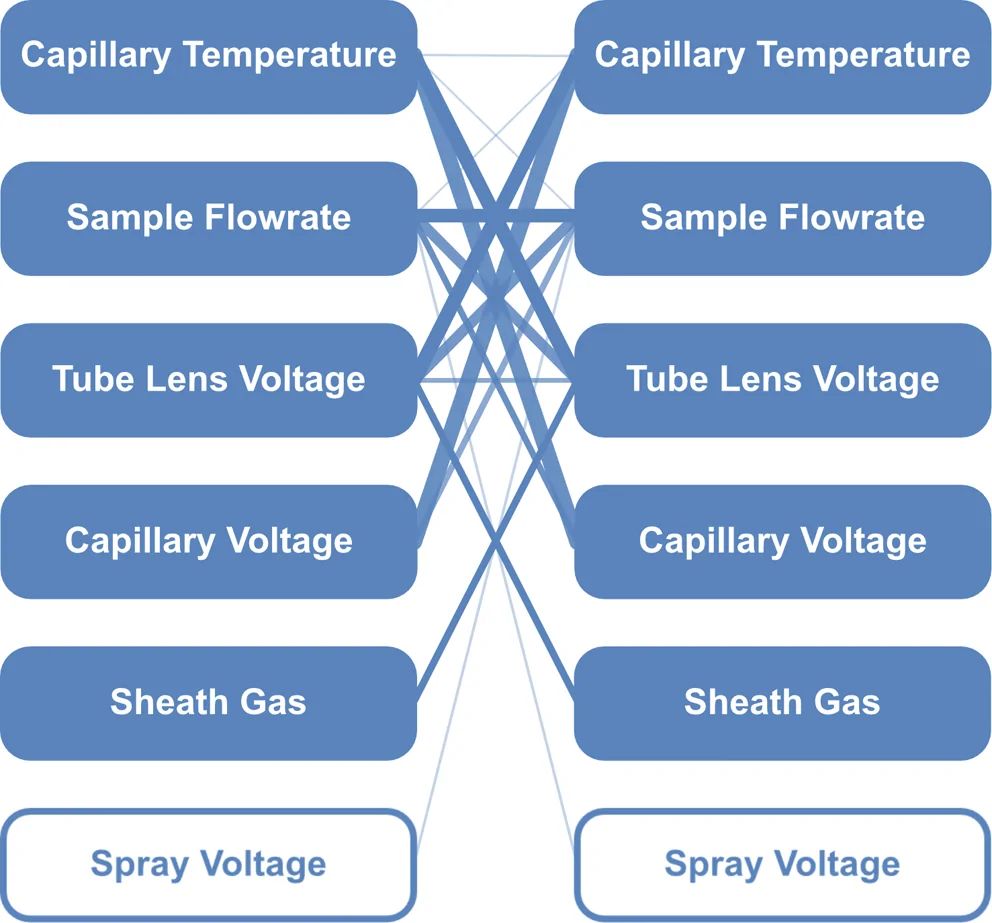
Schematic representation of statistically significant interaction and quadratic effects in the fitted model. Nodes represent ESI source parameters ordered by decreasing main-effect strength; connecting lines indicate significant interaction terms, with line thickness scaled to statistical significance (logworth). Full model details are provided in the Supporting Information.

The presence of these higher-order terms indicates that the internal energy response is not strictly linear across the investigated parameter space. In particular, the quadratic dependence on tube-lens voltage reflects curvature in the activation profile that persists even after restriction to the energy-sensitive regime. Although measurements beyond the tipping point were excluded, tube-lens gradients inherently couple changes in activation efficiency with changes in ion transmission. Because transmission does not vary independently or monotonically across the gradient, a gradual overlap between activation-dominated and transmission-influenced behavior remains unavoidable. Residual coupling between these effects may therefore contribute to the observed nonlinearity.

To visualize the nonlinearity, we examined the model-predicted internal energy profiles as a function of the tube-lens voltage at low and high sample flow rates (Figure 8). This representation explicitly separates the flow-dependent baseline internal energy shift from the incremental energy gain induced by variation of a single source parameter.

**Figure 8:**
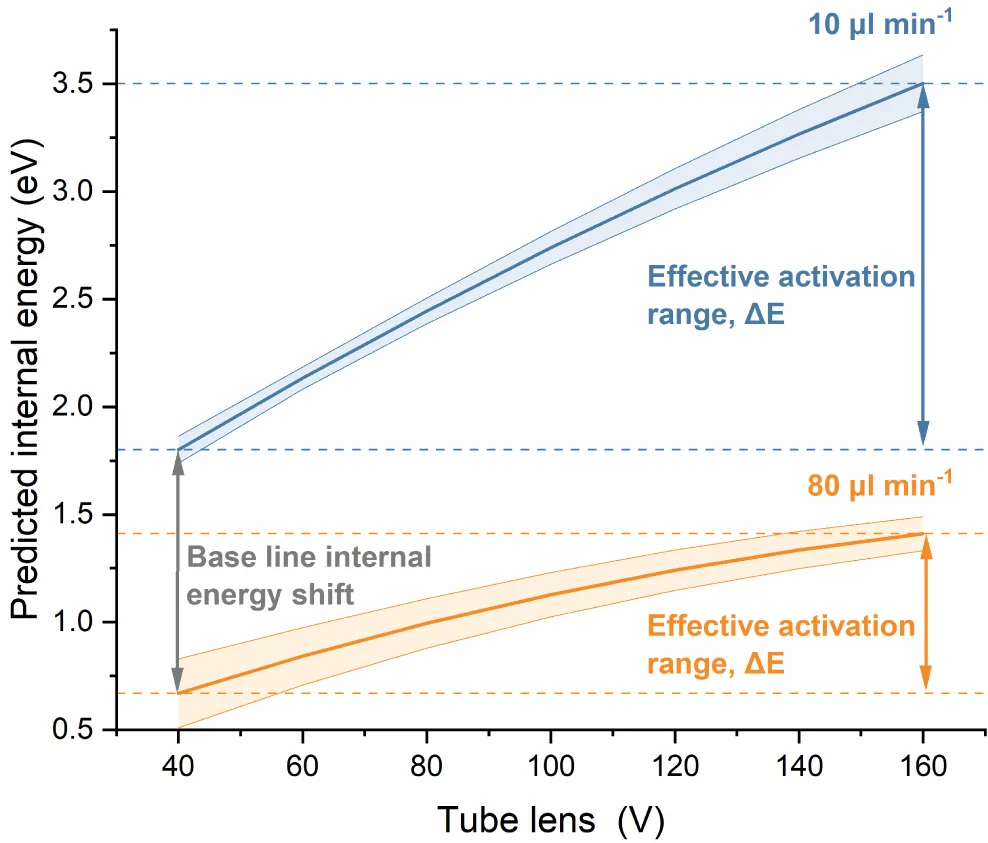
Model-predicted mean internal energy as a function of tube-lens voltage at low (10 μL min^–1^, in blue) and high (80 μL min^–1^, in orange) sample flow rates. Shaded regions indicate the 95% confidence interval of the fitted model. Dashed lines and arrows illustrate the effective energy activation range (ΔE), defined as the change in predicted internal energy over the investigated tube-lens voltage range at fixed flow rate or baseline internal energy shift.

Strikingly, ΔE associated with the tube-lens voltage effect on internal energy decreases with increasing flow rate, indicating that higher liquid throughput progressively suppresses the efficiency of electric-field–driven activation. An analogous trend is observed for capillary voltage, consistent with their shared role as postdesolvation electric-field parameters (Figure 9).

**Figure 9:**
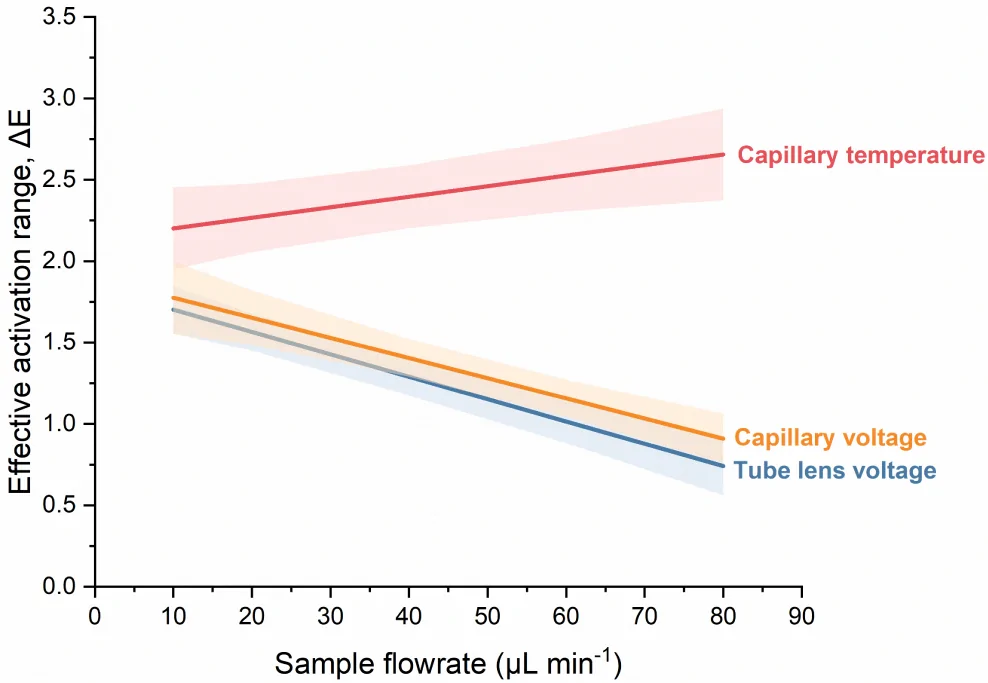
Flow rate dependence of the effective energy activation range (ΔE) associated with tube-lens voltage (blue), capillary voltage (orange), and capillary temperature (red), as predicted by the fitted multivariate model. ΔE is defined as the change in predicted internal energy across the investigated parameter range for each variable, with all other source parameters held constant. Shaded bands indicate propagated 95% mean-confidence intervals derived from end point model predictions.

In contrast to the electric-field-driven activation, the activation energy range associated with capillary temperature exhibits a weak but positive dependence on the sample flow rate (Figure 9). This divergence indicates that increasing the sample flow rate does not uniformly attenuate all activation pathways. Rather, electric-field-mediated activation (tube lens and capillary voltage) is selectively suppressed, whereas thermal activation remains comparatively robust across the explored sample flow range.

Higher sample flow rates not only promote the formation of larger initial droplets but also increase the partial pressure in the source region. Under such conditions, ions may remain transiently shielded by a solvent shell. Hence, collisions in the capillary–skimmer range preferentially complete desolvation rather than impart substantial internal activation. This explanation is consistent with pressure-dependent studies of internal energy distributions and prior observations of reduced fragmentation at elevated flow rates.^3,15,19^ Similar behavior was recently reported by Asakawa et al., who demonstrated that increased fluid and gas throughput in a high-pressure ESI source increased the local pressure in the first vacuum stage and resulted in reduced ion internal energies.^18^ Although the experimental variable investigated here is the sample flow rate rather than source pressure itself, the observed trends are consistent with the same underlying mechanism of pressure- and collision-mediated collisional cooling during ion transfer.

Within this framework, the differing behavior of thermal and electric-field activation reflects a shift in the balance between droplet-phase energy dissipation and postdesolvation energy deposition, which can be quantified experimentally through a model-derived effective activation energy range Δ*E*. The magnitude of this effect is expected to depend on source geometry and ion-transfer conditions.^15^ While the trends described here were consistently observed on the Thermo ESI platforms used in this study, the effects of the flow on the ion activation during ion transfer are likely generalizable.

Intrigued by the strong influence of sample flow rate on internal energy, we investigated whether this effect arises from the average sample flow rate or from time-dependent perturbations in the flow. To probe this, the scan rate of the mass spectrometer was increased to 12.8 Hz (0.078 s per scan), and the syringe pump was operated in a pulsed flow regime by adjusting the microstepping settings. Stepper motors move in discrete steps that can be electronically subdivided (microstepping) to smooth the motion; however, when operated in full-step mode, the resulting flow becomes periodic.

Figure 10 shows the internal energy as a function of time when the syringe pump is operated at a flow rate of 25 μL min^–1^ using full-step operation (1 microstep, ∼ 0.42 μL/step). As shown in Figure 10, the mass spectrometer is able to sample internal energies at different phases of the flow pulse (Figure 10, bottom). Because the mass spectrometer acquisition rate is substantially faster than the pump stepping frequency, multiple measurements are obtained within each pulse cycle, allowing the internal energy to be sampled across the flow perturbation. The pulsed flow profile was independently measured offline using a flow sensor (Figure 10, top) and aligned with the MS signal. Both the total ion current (TIC) and the internal energy oscillate with the same periodicity but are phase-shifted relative to each other. Hence, the temporal structure of the flow induced by the syringe pump directly translates into fluctuations in the internal energy of generated ions.

**Figure 10:**
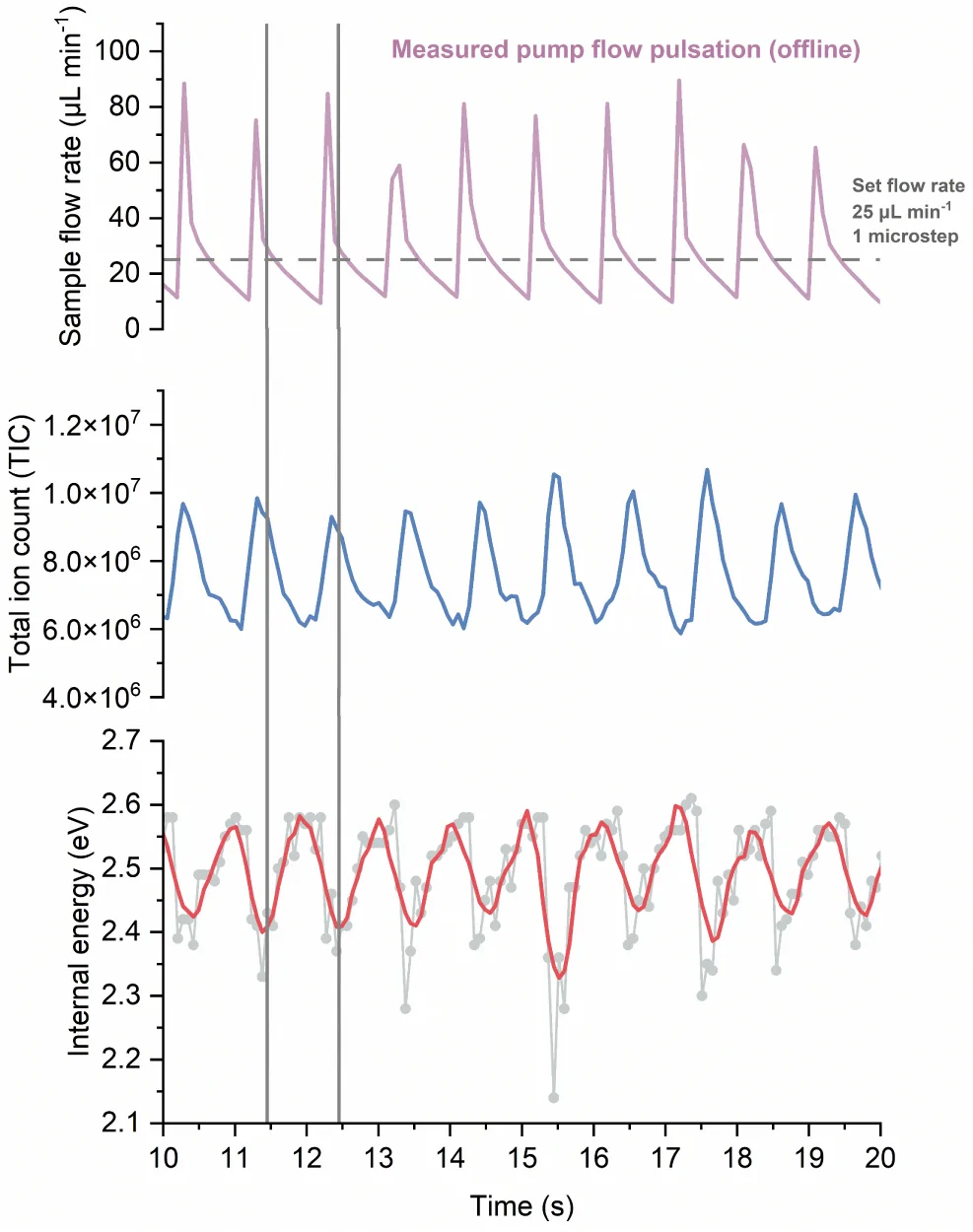
Time-resolved response of ion signal and internal energy to periodic flow perturbations. The syringe pump was operated at a flow rate of 25 μL min^–1^ in full-step mode (1 microstep), generating periodic flow pulses with a period of ∼1 s and an estimated step volume of ∼0.4 μL. The pulsatile flow profile (top) was independently measured in an offline experiment and manually aligned to the total ion current (TIC) trace. The TIC (middle) and the internal energy derived from thermometer ions (bottom) are plotted as a function of time. Both signals oscillate at the pump stepping frequency, demonstrating that the internal energy responds to rapid variations in sample flow rate. Gray points represent the raw internal energy values, while the red line shows a Savitzky–Golay smoothed trace.

Both the mean internal energy and the relationship between sample flow rate and internal energy remain unchanged when different microstepping configurations are used (higher sample flow rate → lower internal energy) (Figure S9). Furthermore, the periodic modulation in internal energy induced by the pulsed flow can be recovered using FFT analysis for pulsation frequencies up to 3.18 Hz (Figure S10). Above these pulsation frequencies, the acquisition rate of the mass spectrometer becomes insufficient to resolve the energy distribution.

Beyond establishing the energetic-control framework that forms the primary objective of this work, the resulting model also suggests a practical analytical application by enabling thermometer ions to serve as real-time reporters of sample flow rate. Although the full response exhibits curvature, well-defined local linear regions can be identified under fixed source conditions and used for calibration (Figure 11).

**Figure 11:**
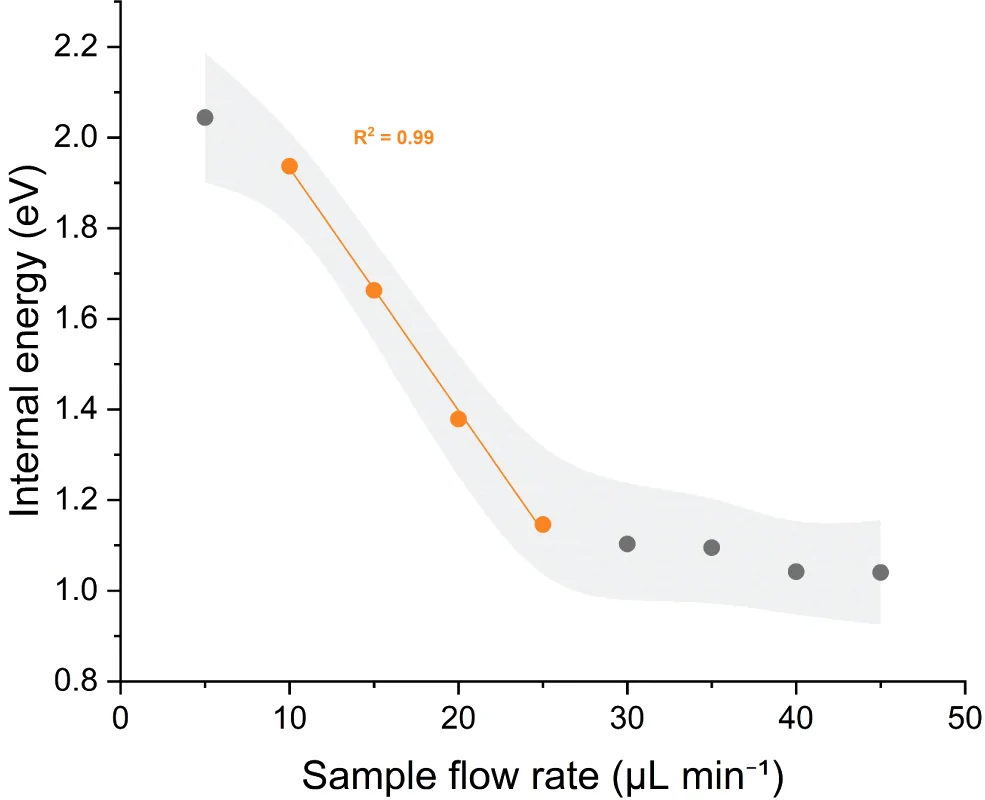
Identification of a locally linear regime in the relationship between sample flow rate and internal energy. The highlighted region (orange) shows the calibration range useful for flow rate estimation.

Within this region, the thermometer-ion-derived internal energy provides a direct indicator of the effective flow rate delivered to the electrospray source. This capability may be particularly useful during reaction monitoring in overpressure-driven or split-flow systems.^8,30–32^ In such configurations, the use of back-pressure regulators and temperature-controlled reactors can make the effective flow reaching the mass spectrometer difficult to determine accurately, and uncertainties in tubing resistance, solvent viscosity, and dynamic pressure conditions can render simple Poiseuille-based estimates unreliable.^33^ Conventional inline flow sensors are frequently unsuitable due to chemical compatibility constraints, additional dead volume, or the presence of electronics in the liquid path near the high-voltage electrospray interface. In contrast, thermometer ions provide an intrinsic probe that requires no additional hardware and can be monitored directly in the mass spectrum. Although the calibration is specific to the source conditions and the selected thermometer-ion set, the local linear response enables straightforward estimation of relative flow rates or dilution ratios in electrospray-based experiments.

The source-specific nature of this response is further illustrated by preliminary experiments performed on additional electrospray source geometries (Figure S11). While the Thermo TSQ Classic 7000 showed a clear sample flow rate dependence in the energetic response, essentially no measurable energetic response was observed with a Bruker Compact Q-TOF instrument under the investigated conditions. Although these measurements do not constitute a complete multivariate characterization, they demonstrate that energetic responses to changing source conditions need not be universal across instrument platforms. Rather than weakening the present framework, these observations reinforce its central premise: the energetic landscape should be established for each source configuration individually before interpreting source-dependent measurements.

## Conclusions

This work presents a practical framework for constructing a source-specific energetic control for electrospray ionization experiments. By mapping combinations of source parameters onto a relative predicted mean internal-energy axis, the framework provides an independent energetic reference that can accompany measurements in which source conditions are intentionally varied. Rather than representing an absolute thermodynamic calibration, the energetic axis serves as a consistent operational coordinate for correlating energetic changes with experimentally observed transformations within a defined source configuration.

Application of this framework revealed that, despite the inherent complexity of the electrospray source, its energetic response is sufficiently systematic to be described by a predictive multivariate model. Sample flow rate emerged as a dominant determinant of ion activation, exhibiting an influence comparable to that of conventional activation parameters such as tube-lens voltage and capillary temperature. The observed flow rate dependence provides a mechanistic explanation for numerous previously reported flow-dependent phenomena in electrospray-based experiments, including changes in fragmentation propensity,^34^ adduction and speciation patterns,^14^ and apparent reaction yields^10^ or equilibrium constants.^16^ Furthermore, the tube-lens gradient experiments demonstrated that differential precursor and fragment transmission can produce misleading energetic trends, emphasizing that transmission-distorted operating regimes should be identified and excluded before quantitative energetic interpretation.

Beyond the specific findings obtained for the Thermo LTQ source, this work highlights a broader methodological consideration. The energetic consequences of changing source conditions should not be interpreted from isolated measurements performed at arbitrarily selected operating points. Instead, the selected operating conditions should first be validated within a physically interpretable energetic regime. The framework presented here therefore enables the energetic softness or hardness of an ESI experiment to be quantitatively established rather than qualitatively assumed, providing the energetic context required to distinguish source-induced effects from genuine chemical transformations. As ESI-MS continues to evolve from a technique for molecular detection into a platform for probing chemical reactivity, independently establishing this energetic landscape is critical.

## Supplementary Material



## Notes

Instrumentation; design of experiments; complete experimental design matrix; additional discussion of transmission effects and data considerations; model development and statistical validation; regression statistics, parameter estimates, ANOVA tables, residual analyses, and goodness-of-fit diagnostics for the multivariate models; supplementary flow rate pulsation and microstepping experiments; comparison of energetic responses across electrospray source designs; references (PDF)

## Abbreviations

ESI: electrospray ionization
MS: mass spectrometry
CID: collision-induced dissociation
DoE: design of experiments
SY: survival yield
BDE: bond dissociation energy
FFT: Fast Fourier transform
TIC: total ion current
RMSE: root-mean-square error
