# Supplementary material for: An Energetic-Control Framework for Electrospray Ionization Using Thermometer Ions
Source: J Am Soc Mass Spectrom. 2026 Oct 1;37(11):2678–87. doi: 10.1021/jasms.6c00109 (PMC13741910; doi:10.1021/jasms.6c00109)
Supplement: Supplementary file 1 [file js6c00109_si_001.pdf]

SUPPORTING INFORMATION

An Energetic-Control Framework for Electrospray Ionization Using Thermometer Ions

Max T.G.M. Derks, and Prof. Dr. Jana Roithová*

Institute for Molecules and Materials, Radboud University, Heyendaalseweg 135, 6525 AJ Nijmegen, The Netherlands

Email: jana.roithova@ru.nl

Supplementary information

Contents

Sample Preparation........ 3
Instrumentation........ 4
Data considerations........ 5
Model design........ 7
Model statistics – Pre tipping filtered model........ 9
Model statistics – Pre tipping and within BDE window filtered model........ 12
References........ 18

Sample Preparation

The thermometer ions used in the analysis include *p*-H,H, *p*-OMe,OMe, *p*-Me,Me, *p*-NO_2, *p*-CN p-CH_3, *p*-OCH_3, and p-F derivatives. These compounds were either available from previous work in our laboratory or synthesized according to previously reported literature procedures.[1,2]

Stock solutions (1 mg mL^{-1}) of each thermometer ion (benzylpyridinium and benzhydrylpyridinium derivatives) were prepared in methanol–water (1:1, v/v).

For each experiment, 100 µL of the respective stock solutions were combined and diluted to a final volume of 10 mL with methanol–water (1:1, v/v) to prepare the working solution.

The thermometer ions included in the analysis were: *p*-H,H; *p*-OMe,OMe; *p*-NO_2; *p*-CH_3; *p*-OCH_3; and *p*-F. Additional derivatives (*p*-Me,Me and *p*-CN) were included in the prepared mixture but excluded from data analysis due to isobaric overlap in their *m/z* values.

The thermometer ions included in the analysis of the time-resolved flow pulsation measurements included : *p*-H,H; *p*-OMe,OMe; p-Me,Me; p-NO_2; *p*-CH_3; *p*-OCH_3; and *p*-F and after combination of the stock solutions it was further diluted with MeCN instead of methanol-water.

Table S1*: Thermometer ions used in this analysis of the DoE study [1]values obtained from ref 1*

Thermometer ion	Parent mass	Fragment mass	E_0 (eV), CCSD(T)[1]
**Benzylpyridinium**			
p-CH_3	184.1	105	2.267
p-OCH_3	200.1	121	1.86
p-F	188.1	109	2.392
p-NO_2	215, 169[b]	136	2.843
**Benzhydrylpyridinium**			
p-H,H	246.1	167	1.74
p-OCH_3,OCH_3	305.5	227	1.18

Instrumentation

All measurements included in the design of experiments were performed on an LTQ XL mass spectrometer equipped with an Ion Max API Source and operated in electrospray ionization (ESI) mode. The flowrate calibration experiments were performed on a triple quadrupole mass spectrometer (TSQ Classic) equipped with an electrospray ionization source. Control experiments on a different mass spectrometer were performed on a at Bruker Compact QTOF mass spectrometer with an electrospray ionization source.

The resulting thermometer-ion mixture was loaded into a 1002 TLL syringe (Hamilton, Reno, NV, USA) and samples were infused using a syringe pump (Labm8 M8003, Labm8, Netherlands). The stepper motor was operated at 16× microstepping with driver interpolation to 256 microsteps, unless otherwise stated. Flow-rate pulsations were independently measured offline using a Sensirion SLF-0600 flow sensor with a sampling interval of 100 ms.

The distance between the electrospray needle and the ion transfer capillary entrance was kept constant throughout all experiments and set to the maximum available distance.

No auxiliary gas or sheath liquid were used throughout the experiments and the other source parameters were investigated according to the DoE table, table 2 summarizes the parameters and their levels, while table 3 provides the complete list of conditions tested. The run order represents the actual order in which the experiments were executed, please note that the parameter capillary temperature and sample flowrate were blocked from the randomization to improve experimental time efficiency.

Table S2: *Parameters and levels tested in the design of Experiments*

Parameter	Levels
**Spray Voltage (kV)**	3.5, 5
**Temperature (°C)**	150, 220, 300
**Sheath Gas (arb. units)**	5, 20
**Capillary Voltage (V)**	40, 100
**Sample Flowrate (µL min^{-1})**	5, 25, 80
**Tube Lens Voltage (V)**	40 – 240 (20V increments)

Data considerations

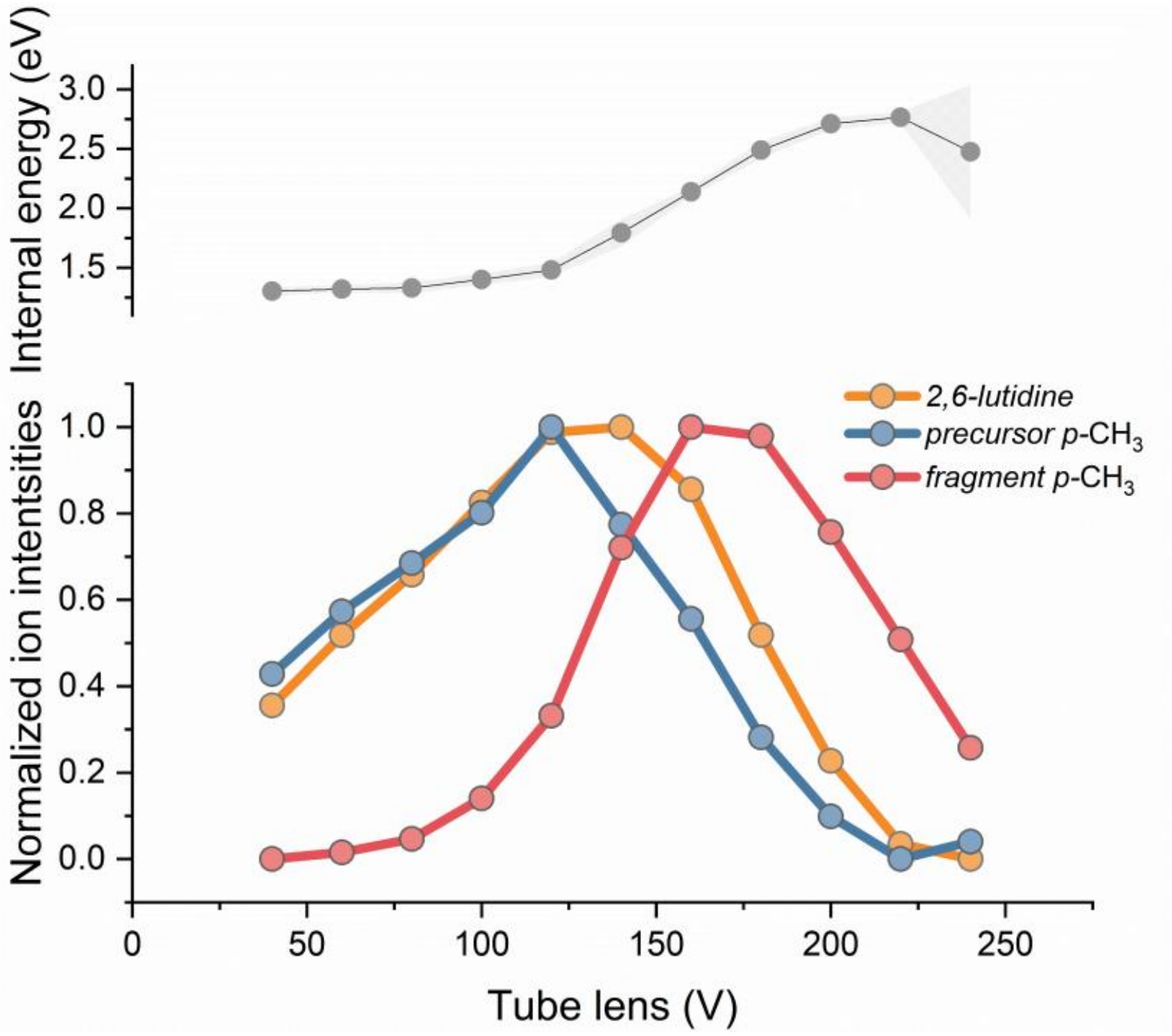

Figure S1: *Influence of tube-lens voltage on ion transmission and apparent internal energy. The upper panel shows the internal energy derived from thermometer ions as a function of tube-lens voltage. The lower panel displays normalized ion intensities for the precursor ion (p-CH_3 benzylpyridinium), its fragment ion, and the transmission probe 2,6-lutidine. As the tube-lens voltage increases, the precursor ion intensity decreases while fragment intensity increases due to collision-induced dissociation. At higher voltages, a decline in transmission of the reference ion (2,6-lutidine) indicates reduced ion transmission efficiency. These results illustrate that apparent changes in internal energy at high tube-lens settings can be influenced by transmission losses rather than purely by energetic activation.*

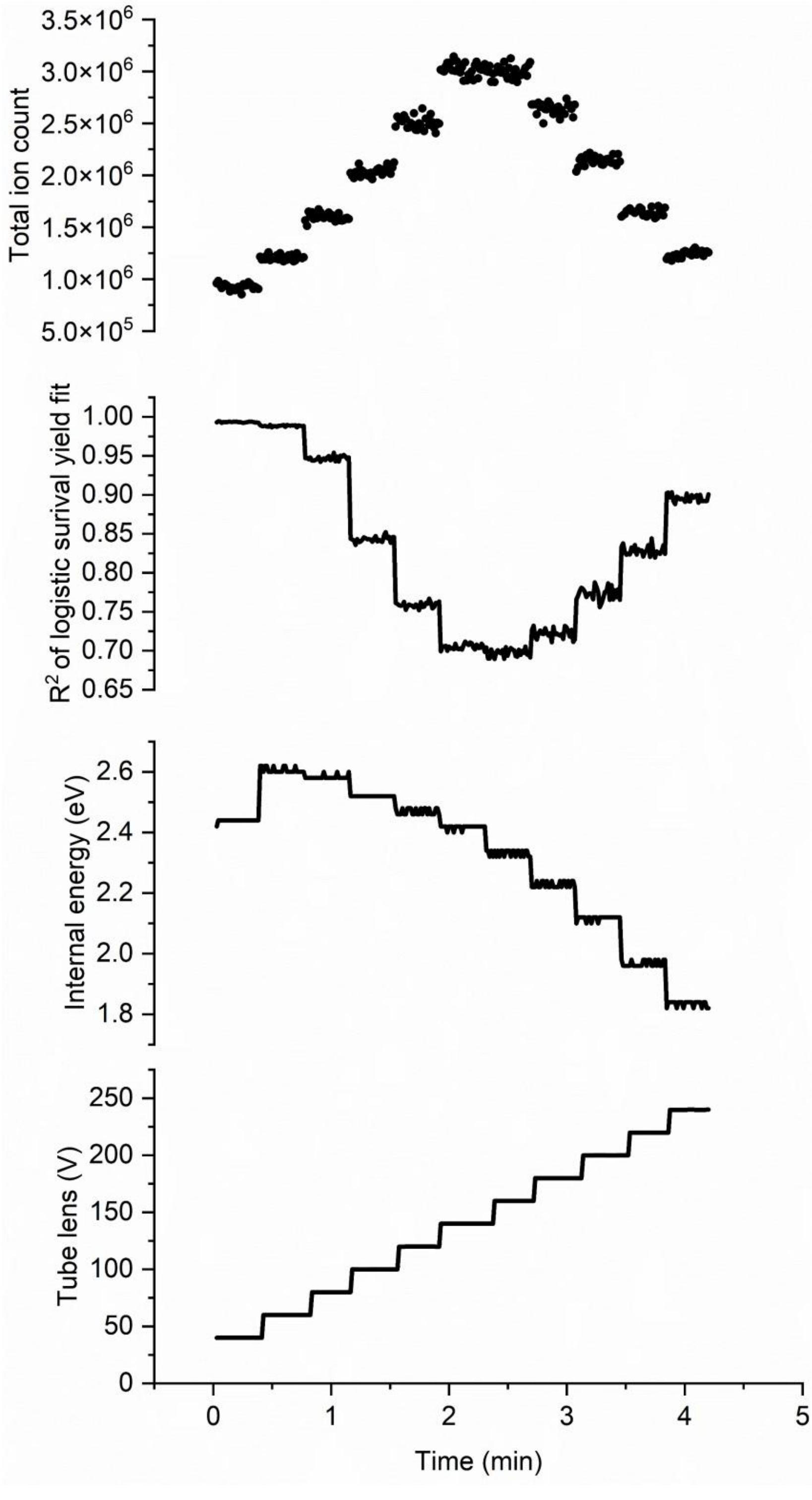

Figure S2: *Time traces of tube lens voltage, extracted internal energy, goodness-of-fit (R^2) of the logistic survival yield model, and total ion count (TIC) during a representative tube lens gradient (DoE run 27). Although TIC and R^2 vary systematically with tube lens voltage, neither metric provides a clear criterion to distinguish between regimes dominated by physical activation and those affected by transmission losses.*

Model design

Table S3: *Overview of tested source conditions in the Design of experiments, each condition was run as a Tube Lens gradient*

Gradient Run #	SheathGas	Spray Voltage (kV)	Capillary temperature (°C)	Capillary Voltage (V)	Sample Flowrate (µl min^{-1})
**1**	5	5	150	100	5
**2**	20	3.5	150	40	5
**3**	5	3.5	150	100	5
**4**	5	5	150	40	5
**5**	20	5	150	100	5
**6**	5	3.5	150	40	5
**7**	20	5	150	40	5
**8**	20	3.5	150	100	5
**9**	20	5	150	40	25
**10**	20	5	150	100	25
**11**	20	3.5	150	40	25
**12**	20	3.5	150	100	25
**13**	5	5	150	40	25
**14**	5	5	150	100	25
**15**	5	3.5	150	40	25
**16**	5	3.5	150	100	25
**17**	20	5	150	40	80
**18**	20	5	150	100	80
**19**	20	3.5	150	40	80
**20**	5	5	150	40	80
**21**	20	3.5	150	100	80
**22**	5	3.5	150	40	80
**23**	5	5	150	100	80
**24**	5	3.5	150	100	80
**25**	20	3.5	225	100	5
**26**	20	5	225	100	5
**27**	5	3.5	225	100	5
**28**	5	5	225	100	5
**29**	5	3.5	225	40	5
**30**	20	3.5	225	40	5
**31**	20	5	225	40	5
**32**	5	5	225	40	5
**33**	5	3.5	225	100	25
**34**	5	5	225	100	25
**35**	20	3.5	225	100	25
**36**	20	3.5	225	40	25
**37**	5	3.5	225	40	25
**38**	20	5	225	100	25
**39**	5	5	225	40	25
**40**	20	5	225	40	25

**41**	20	5	225	40	80
**42**	5	3.5	225	100	80
**43**	5	3.5	225	40	80
**44**	20	3.5	225	100	80
**45**	5	5	225	40	80
**46**	5	5	225	100	80
**47**	20	3.5	225	40	80
**48**	20	5	225	100	80
**49**	20	3.5	300	40	5
**50**	20	5	300	100	5
**51**	20	5	300	40	5
**52**	5	3.5	300	40	5
**53**	5	3.5	300	100	5
**54**	20	3.5	300	100	5
**55**	5	5	300	40	5
**56**	5	5	300	100	5
**57**	20	3.5	300	40	25
**58**	20	5	300	40	25
**59**	5	3.5	300	40	25
**60**	5	5	300	40	25
**61**	5	3.5	300	100	25
**62**	20	3.5	300	100	25
**63**	20	5	300	100	25
**64**	5	5	300	100	25
**65**	5	3.5	300	40	80
**66**	20	5	300	100	80
**67**	5	5	300	100	80
**68**	5	3.5	300	100	80
**69**	5	5	300	40	80
**70**	20	5	300	40	80
**71**	20	3.5	300	40	80
**72**	20	3.5	300	100	80

Model statistics – Pre tipping filtered model

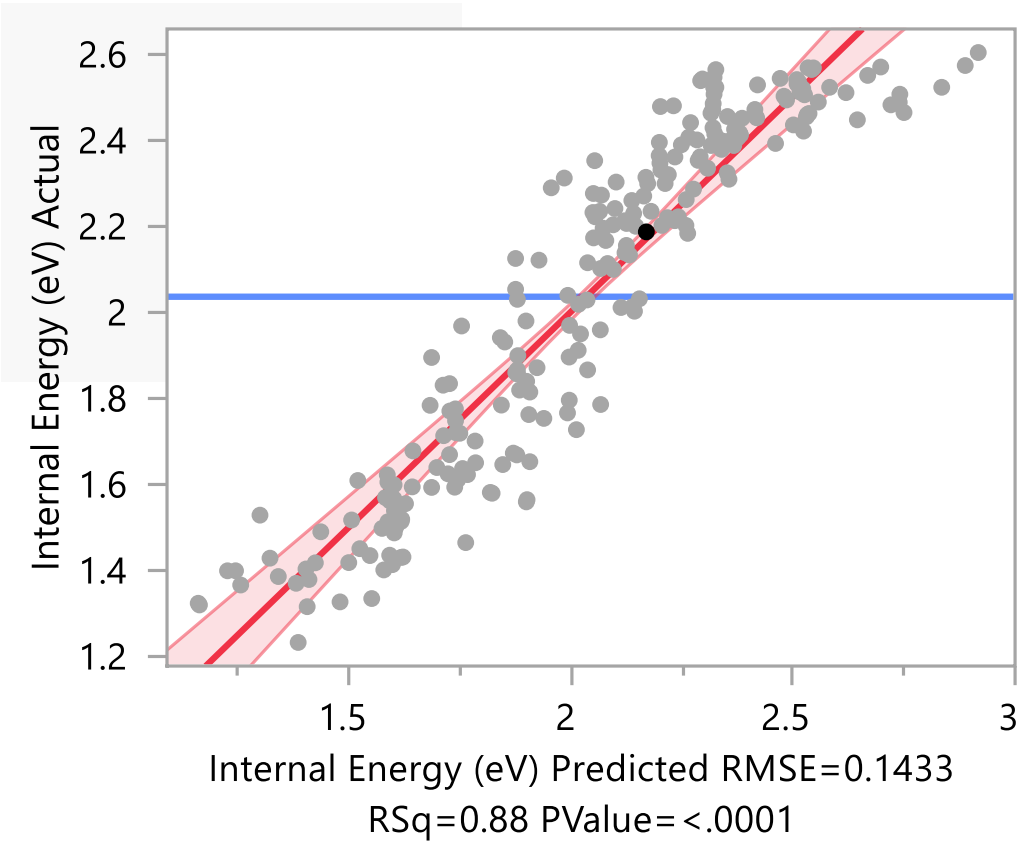

Figure S3: *Actual versus predicted relative mean internal energy for the multivariate model fitted after excluding the pre-tipping region. The solid line represents the ideal 1:1 agreement, while the shaded bands indicate the confidence interval of the fit. The model captures the measured energetic response with good agreement (R^2 = 0.88, RMSE = 0.143 eV).*

Table S4*: Effect summary of the multivariate model constructed after exclusion of the pre-tipping region. Effects are ranked according to LogWorth (−log10 P-value), highlighting the relative importance of individual source parameters and statistically significant interaction terms.*

**Source**	**Logworth**	**PValue**
Capillary Temperature	90.059	0.00000
Sample flowrate	82.913	0.00000
Capillary Voltage	72.920	0.00000
Tube Lens Voltage	63.177	0.00000
Capillary Temperature*Sample flowrate	44.391	0.00000
Sheath Gas Flowrate	36.583	0.00000
Tube Lens Voltage*Capillary Voltage	22.680	0.00000
Sample flowrate*Sample flowrate	19.511	0.00000
Capillary Temperature*Capillary Temperature	18.031	0.00000
Capillary Voltage*Sample flowrate	12.049	0.00000
Tube Lens Voltage*Tube Lens Voltage	11.760	0.00000
Tube Lens Voltage*Sheath Gas Flowrate	7.791	0.00000
SprayVoltage*Sample flowrate	1.957	0.01104
Sheath Gas Flowrate*Capillary Voltage	1.837	0.01457
SprayVoltage	0.613	0.24377 ^

Table S5: *Lack-of-fit analysis for the multivariate model after exclusion of the pre-tipping region. The non-significant lack of fit (P = 0.4407) indicates that the selected model adequately describes the experimental data.*

Source	DF	Sum of Squares	Mean Square	F Ratio
Lack Of Fit	209	4.3064665	0.020605	1.7161
Pure Error	2	0.0240142	0.012007	**Prob > F**
Total Error	211	4.3304807		0.4407
				**Max RSq**
				0.9993

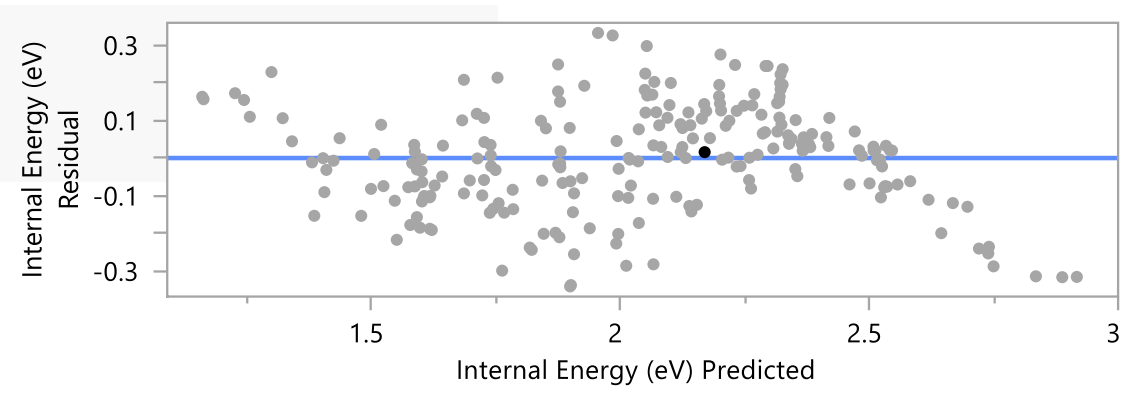

Figure S4: *Residuals plotted against predicted relative mean internal energy for the pre-tipping filtered model. The absence of strong systematic trends indicates that the model adequately captures the dependence of the response on the investigated source parameters.*

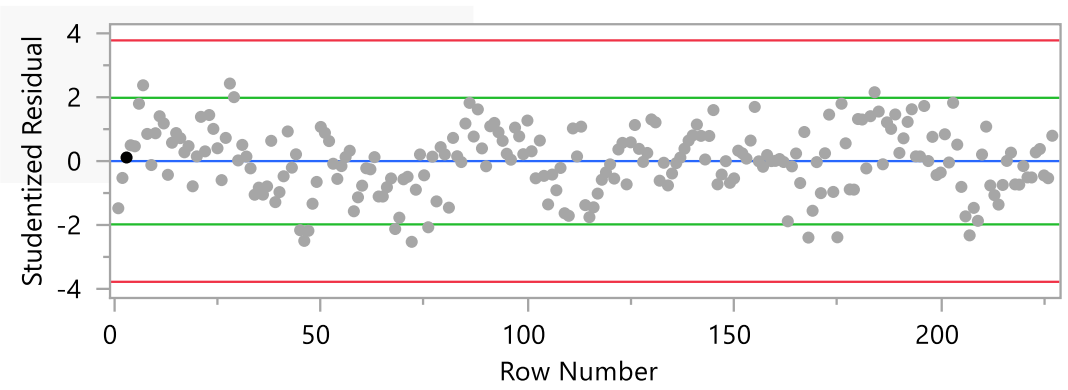

Figure S5: *Studentized residuals for the pre-tipping filtered model. Red lines indicate the simultaneous Bonferroni limits and green lines the individual confidence limits. No substantial outliers are observed*

Table S6: *Summary statistics of the multivariate model after exclusion of the pre-tipping region, including the coefficient of determination (R^2), adjusted R^2, root mean square error (RMSE), and information criteria.*

RSquare	0.878077
RSquare Adj	0.86941
Root Mean Square Error	0.143261
Mean of Response	2.034407
Observations (or Sum Wgts)	227

AICc	-217.628
BIC	-162.332

Table S7: *Parameter estimates for the multivariate model after exclusion of the pre-tipping region. Linear, quadratic, and interaction coefficients are reported together with their standard errors, t-ratios, and statistical significance.*

Term	Estimate	Std Error	t Ratio	Prob>|t|
Intercept	-1.857785	0.128446	-14.46	<.0001*
Tube Lens Voltage	0.0084116	0.000342	24.60	<.0001*
Sheath Gas Flowrate	0.0222319	0.001416	15.70	<.0001*
SprayVoltage	0.0149822	0.012818	1.17	0.2438
Capillary Temperature	0.0113351	0.00032	35.43	<.0001*
Capillary Voltage	0.0123855	0.000438	28.25	<.0001*
Sample flowrate	-0.025564	0.000791	-32.31	<.0001*
(Tube Lens Voltage-112.775)*(Tube Lens Voltage-112.775)	-3.37e-5	4.492e-6	-7.50	<.0001*
(Tube Lens Voltage-112.775)*(Sheath Gas Flowrate-12.4009)	0.0001506	2.563e-5	5.88	<.0001*
(Capillary Temperature-224.339)*(Capillary Temperature-224.339)	-4.333e-5	4.447e-6	-9.74	<.0001*
(Tube Lens Voltage-112.775)*(Capillary Voltage-68.8106)	-8.871e-5	7.863e-6	-11.28	<.0001*
(Sheath Gas Flowrate-12.4009)*(Capillary Voltage-68.8106)	0.0001085	0.000044	2.46	0.0146*
(SprayVoltage-4.26652)*(Sample flowrate-36.0793)	0.0011112	0.000433	2.56	0.0110*
(Capillary Temperature-224.339)*(Sample flowrate-36.0793)	0.0001865	1.025e-5	18.20	<.0001*
(Capillary Voltage-68.8106)*(Sample flowrate-36.0793)	0.000091	0.000012	7.61	<.0001*
(Sample flowrate-36.0793)*(Sample flowrate-36.0793)	0.0002207	2.155e-5	10.24	<.0001*

Table S8: *Statistical significance of individual model terms for the pre-tipping filtered model determined by analysis of variance (ANOVA). Significant linear, interaction, and quadratic effects are observed for several source parameters.*

Source	Nparm	DF	Sum of Squares	F Ratio	Prob > F
Tube Lens Voltage	1	1	12.419942	605.1540	<.0001*
Sheath Gas Flowrate	1	1	5.058222	246.4587	<.0001*
SprayVoltage	1	1	0.028041	1.3663	0.2438
Capillary Temperature	1	1	25.767690	1255.515	<.0001*
Capillary Voltage	1	1	16.383001	798.2516	<.0001*
Sample flowrate	1	1	21.424569	1043.899	<.0001*
Tube Lens Voltage*Tube Lens Voltage	1	1	1.154741	56.2640	<.0001*
Tube Lens Voltage*Sheath Gas Flowrate	1	1	0.708748	34.5333	<.0001*
Capillary Temperature*Capillary Temperature	1	1	1.948080	94.9190	<.0001*
Tube Lens Voltage*Capillary Voltage	1	1	2.612422	127.2886	<.0001*
Sheath Gas Flowrate*Capillary Voltage	1	1	0.124523	6.0673	0.0146*
SprayVoltage*Sample flowrate	1	1	0.134939	6.5748	0.0110*
Capillary Temperature*Sample flowrate	1	1	6.796024	331.1321	<.0001*
Capillary Voltage*Sample flowrate	1	1	1.188894	57.9281	<.0001*
Sample flowrate*Sample flowrate	1	1	2.152188	104.8640	<.0001*

Equation S1: *Complete mathematical expression of the reduced multivariate model constructed after exclusion of the post-tipping (transmission-distorted) region. The equation corresponds to the regression model described in the main text and includes all retained linear, quadratic, and interaction terms.*

(-1.85778529941505) + 0.00841157175497202 * :TubeLens_V + 0.0222318596071162 * :SheathGas + 0.014982242510004 * :SprayVoltage_kV + 0.0113350520834268 * :Temp_C + 0.0123854778290845 * :Capillary_V + -0.0255643323013142 * :Sample_Flow_uL_min + -0.0000336978266227646 * (:TubeLens_V - 112.775330396476) * (:TubeLens_V - 112.775330396476) + 0.000150623569194317 * (:SheathGas -12.4008810572687) * (:TubeLens_V - 112.775330396476) + -0.0000433281993938757 * (:Temp_C - 224.339207048458) * (:Temp_C - 224.339207048458) + -0.000088713820872675 * (:Capillary_V - 68.8105726872247) * (:TubeLens_V - 112.775330396476) + 0.000108452468126669 * (:Capillary_V - 68.8105726872247) * (:SheathGas - 12.4008810572687) + 0.00111120215393404 * (:Sample_Flow_uL_min -36.079295154185) * (:SprayVoltage_kV - 4.26651982378855) + 0.000186525884442616 * (:Sample_Flow_uL_min - 36.079295154185) * (:Temp_C - 224.339207048458) +0.0000910089277350636 * (:Sample_Flow_uL_min - 36.079295154185) * (:Capillary_V -68.8105726872247) + 0.000220710906563567 * (:Sample_Flow_uL_min - 36.079295154185) * (:Sample_Flow_uL_min - 36.079295154185)

Model statistics – Pre tipping and within BDE window filtered model

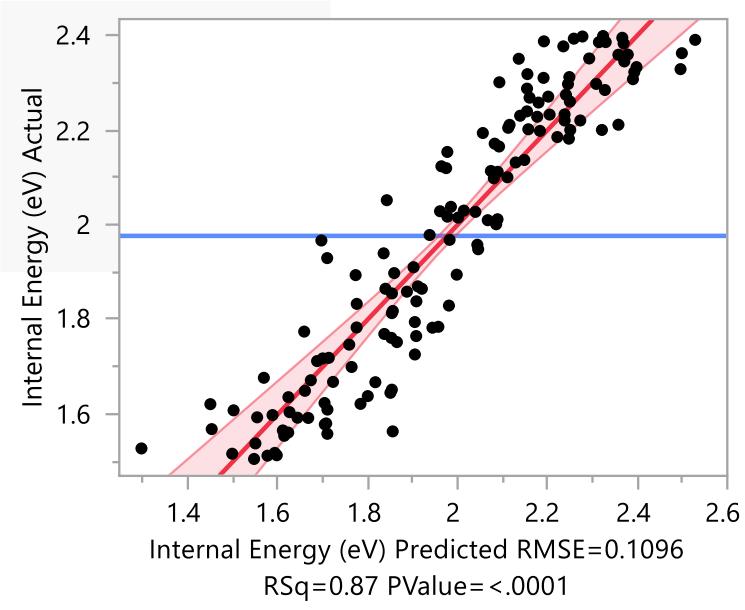

Figure S6: *Actual versus predicted relative mean internal energy for the multivariate model after exclusion of both the pre-tipping region and transmission-distorted measurements outside the selected bond-dissociation-energy window. The resulting model maintains good predictive performance ($R^2 = 0.87$, RMSE = 0.110 eV).*

Table S9*: Effect summary of the multivariate model after exclusion of both the pre-tipping region and measurements outside the selected bond-dissociation-energy window. The ranking illustrates the relative contributions of the investigated source parameters and their interactions to the energetic response.*

Source	Logworth	PValue
Capillary Temperature	52.431	0.00000
Sample Flowrate	50.839	0.00000
Tube Lens Voltage	49.940	0.00000
Capillary Voltage	46.914	0.00000
SheathGas	34.412	0.00000
Capillary Temperature*Capillary Voltage	22.258	0.00000
Tube Lens Voltage*Capillary Temperature	19.691	0.00000
Sample Flowrate*Sample Flowrate	18.477	0.00000
Tube Lens Voltage*Sample Flowrate	14.450	0.00000
Tube Lens Voltage*SheathGas	8.898	0.00000
Capillary Voltage*Sample Flowrate	7.828	0.00000
Tube Lens Voltage*Tube Lens Voltage	5.470	0.00000
Spray Voltage*Sample Flowrate	2.092	0.00809
Capillary Temperature*Capillary Temperature	1.791	0.01619
Capillary Temperature*Sample Flowrate	1.514	0.03065
Spray Voltage	0.398	0.39950 ^

Table S10: *Lack-of-fit analysis for the multivariate model after exclusion of both the pre-tipping region and transmission-distorted measurements outside the selected bond-dissociation-energy window. The non-significant lack of fit (P = 0.6292) indicates an adequate model description of the experimental data.*

Source	DF	Sum of Squares	Mean Square	F Ratio
Lack Of Fit	123	1.4766265	0.012005	0.9998
Pure Error	2	0.0240142	0.012007	**Prob > F**
Total Error	125	1.5006407		0.6292
				**Max RSq**
				0.9979

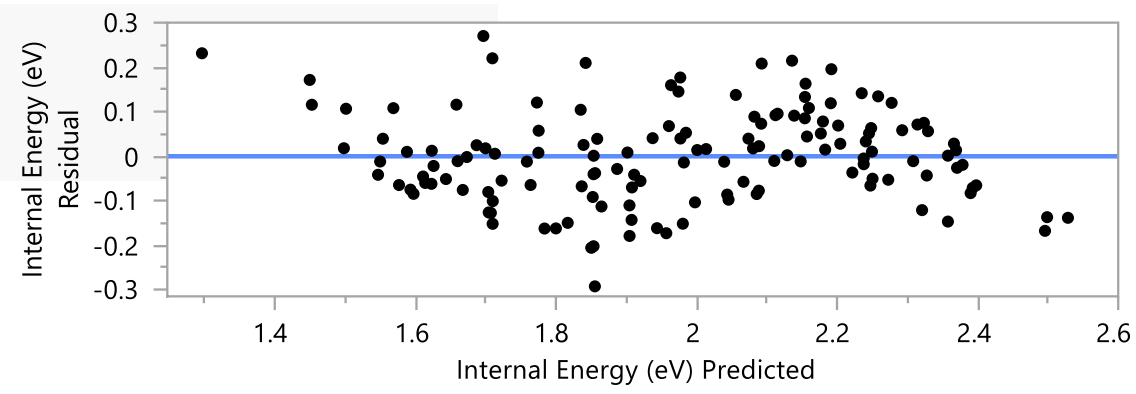

Figure S7: *Residuals plotted against predicted relative mean internal energy for the final multivariate model after exclusion of both the pre-tipping region and transmission-distorted measurements. The residual distribution shows no pronounced systematic dependence on the predicted response.*

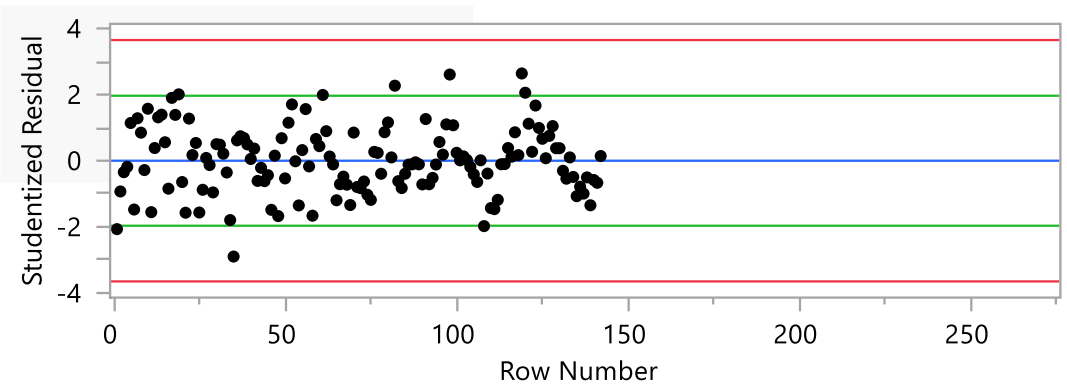

Figure S8: *Studentized residuals for the final multivariate model. Red lines indicate the simultaneous Bonferroni limits and green lines the individual confidence limits. No substantial outliers are observed.*

Table S11: *Summary statistics of the final multivariate model after exclusion of both the pre-tipping region and transmission-distorted measurements outside the selected bond-dissociation-energy window.*

RSquare	0.871573
RSquare Adj	0.855134
Root Mean Square Error	0.109568
Mean of Response	1.976228
Observations (or Sum Wgts)	142
AICc	-201.551
BIC	-153.907

Equation S2: *Complete mathematical expression of the final reduced multivariate model constructed after exclusion of the post-tipping (transmission-distorted) region and restriction of the analysis to the reliable thermometer-ion response range (1.5–2.4 eV). The equation includes all retained linear, quadratic, and interaction terms.*

```
(-2.94161825983824) + 0.0104501826590647 * :TubeLens_V + 0.0265530703260911
 * :SheathGas + 0.0105034056118853 * :SprayVoltage_kV + 0.014604581332719 * :Temp_C
 + 0.0182087390920916 * :Capillary_V + -0.0310334582909699 * :Sample_Flow_uL_min +
-0.0000240445736175301 * (:TubeLens_V - 116.338028169014) * (
:TubeLens_V - 116.338028169014) + 0.000178882741266788 * (:SheathGas
-13.1338028169014) * (:TubeLens_V - 116.338028169014) + 0.0000850962977826689
 * (:Temp_C - 220.246478873239) * (:TubeLens_V - 116.338028169014)
+0.0000172900096262204 * (:Temp_C - 220.246478873239) * (:Temp_C - 220.246478873239)
 + 0.000190409101329818 * (:Capillary_V - 64.5070422535211) * (:Temp_C
-220.246478873239) + -0.000114404163562093 * (:Sample_Flow_uL_min - 36.1267605633803)
* (
:TubeLens_V - 116.338028169014) + 0.00112452550733295 * (:Sample_Flow_uL_min
-36.1267605633803) * (:SprayVoltage_kV - 4.29225352112676) + 0.0000405254405998956
 * (:Sample_Flow_uL_min - 36.1267605633803) * (:Temp_C - 220.246478873239) +
-0.000154620598560261 * (:Sample_Flow_uL_min - 36.1267605633803) * (:Capillary_V
-64.5070422535211) + 0.000341963438673809 * (:Sample_Flow_uL_min - 36.1267605633803)
* (:Sample_Flow_uL_min - 36.1267605633803)
```

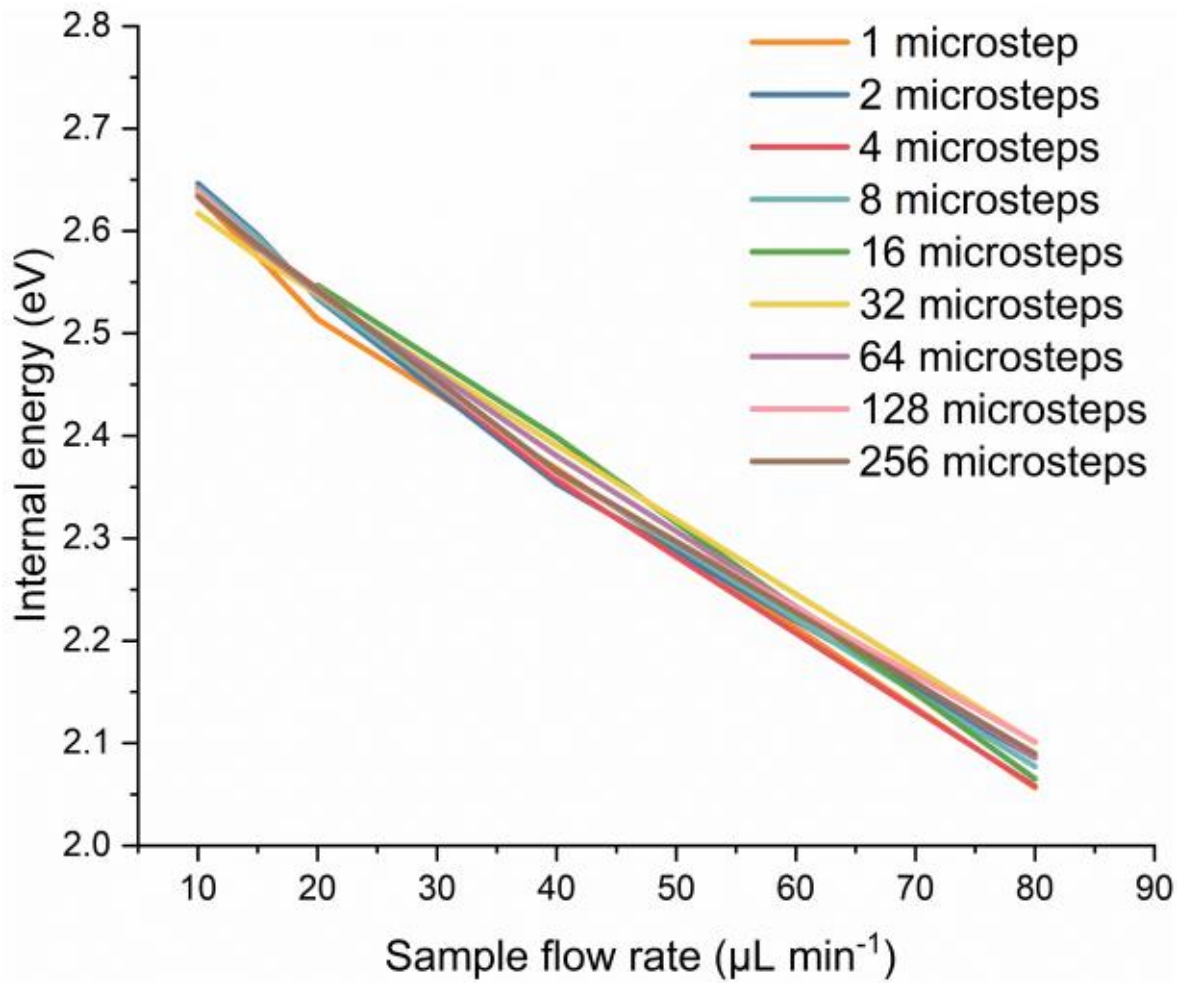

Figure S9. *Dependence of measured internal energy on sample flow rate for different microstepping configurations of the syringe pump. Internal energy values derived from thermometer ions are plotted as a function of flow rate for microstepping settings ranging from full-step operation to 256 microsteps. All datasets follow the same monotonic trend, indicating that the relationship between internal energy and flow rate is independent of the chosen microstepping mode. The microstepping configuration therefore affects the temporal structure of the flow delivery but does not alter the overall flow–energy relationship.*

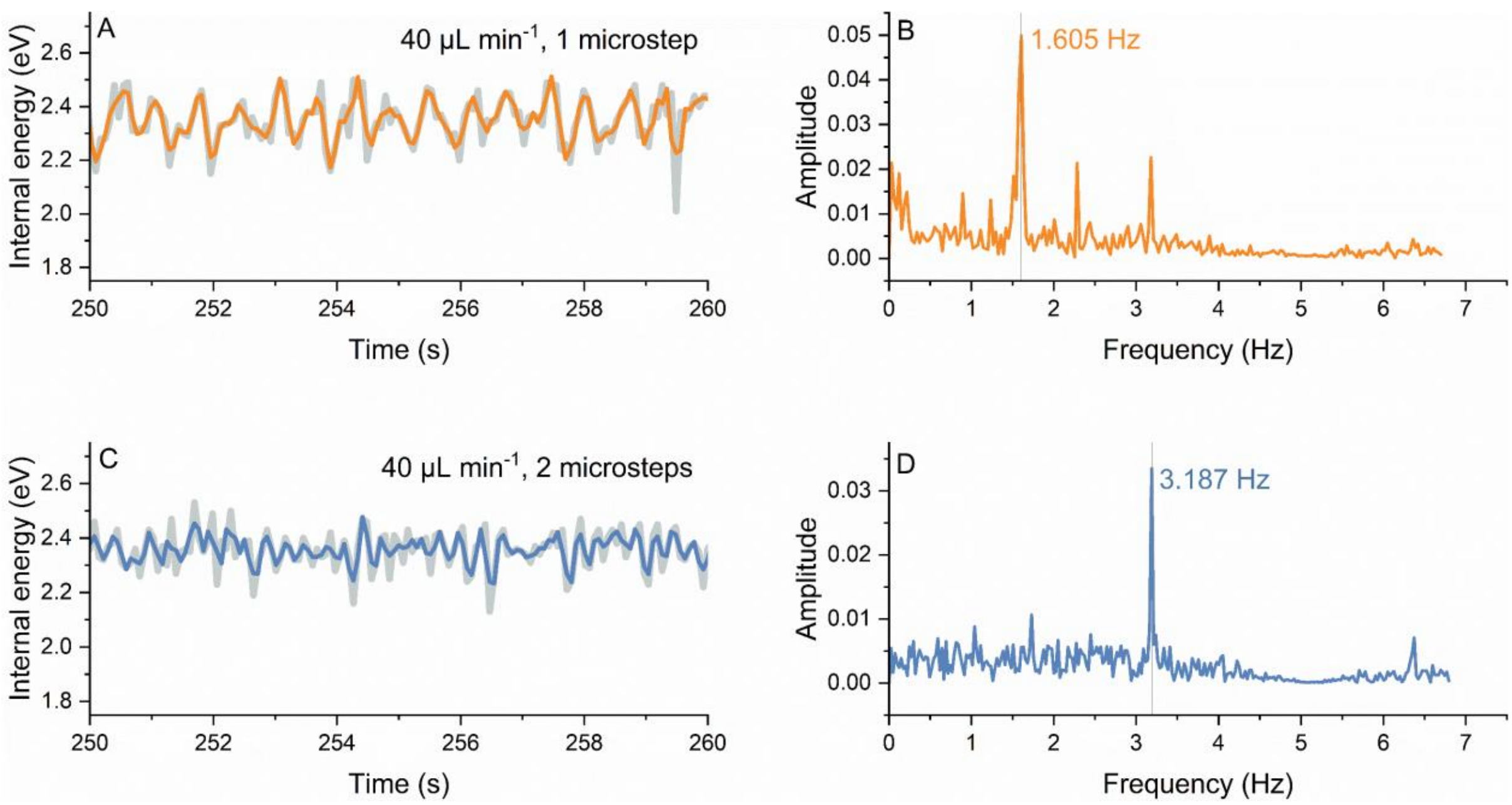

Figure S10. *Detection of syringe-pump stepping frequencies through time-resolved internal-energy measurements. (A) Internal-energy trace recorded at a flow rate of 40 µL min^{-1} using full-step operation (1 microstep per step). (B) Fast Fourier transform (FFT) of the signal in panel A showing a dominant frequency of 1.605 Hz corresponding to the pump stepping frequency. (C) Internal-energy trace recorded at the same flow rate using two microsteps per step. (D) FFT of the signal in panel C showing a dominant frequency of 3.187 Hz. Increasing the microstepping setting increases the stepping frequency while reducing the amplitude of the flow perturbation, demonstrating that internal energy responds directly to the periodic flow modulation generated by the syringe pump.*

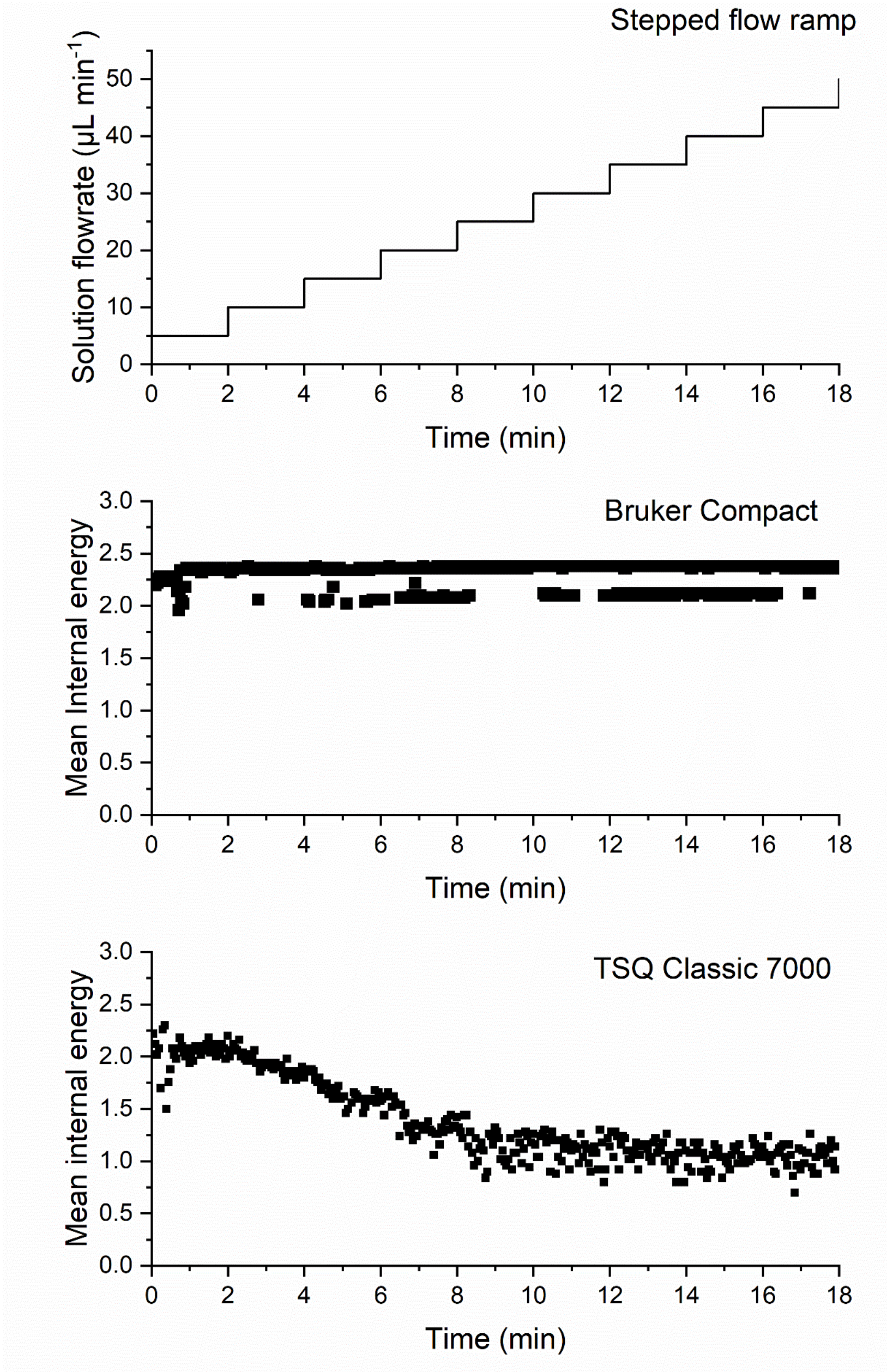

Figure S11: *Relative mean internal energy measured during an identical stepped sample flow-rate ramp on two different electrospray source designs. While a clear flow-rate dependence is observed for the Thermo TSQ Classic 7000, no measurable energetic response is observed for the Bruker Compact under the investigated conditions. These measurements demonstrate that the influence of source parameters on ion activation can differ substantially between instrument platforms.*

References

(1) Rahrt, R.; Auth, T.; Demireva, M.; Armentrout, P. B.; Koszinowski, K. Benzhydrylpyridinium Ions: A New Class of Thermometer Ions for the Characterization of Electrospray-Ionization Mass Spectrometers. *Anal. Chem.* **2019**, *91* (18), 11703–11711. https://doi.org/10.1021/acs.analchem.9b02257.

(2) Rathnayake, M. D.; Weaver, J. D. I. Coupling Photocatalysis and Substitution Chemistry to Expand and Normalize Redox-Active Halides. *Org. Lett.* **2021**, *23* (6), 2036–2041. https://doi.org/10.1021/acs.orglett.1c00173.
